# Calibration-Aware Patient-Level Prediction of Source-Defined MSIMUT Status in Colorectal Cancer from Whole-Slide Histopathology Images Using Foundation-Model Embeddings

**DOI:** 10.3390/diagnostics16203271

**Published:** 2026-10-09

**Authors:** Deniz Özkan Vardar, Necati Vardar

**Affiliations:** 1 Department of Pharmacy Services, Vocational School of Health Services, Lokman Hekim University, Ankara 06510, Turkey; deniz.ozkanvardar@lokmanhekim.edu.tr; 2 Department of Electrical and Electronics Engineering, Faculty of Engineering and Natural Sciences, KTO Karatay University, Konya 42020, Turkey

**Keywords:** source-defined MSIMUT, microsatellite instability-related status, colorectal cancer, whole-slide imaging, computational pathology, foundation model embeddings, probability calibration

## Abstract

**Background/Objectives:** Microsatellite instability (MSI) is clinically important in colorectal cancer, but definitive assessment requires molecular or immunohistochemical testing. This study developed a leakage-safe, calibration-aware patient-level framework for predicting a source-defined MSIMUT category from hematoxylin-and-eosin whole-slide-derived patches using fixed Phikon foundation-model embeddings. **Methods:** The TCGA-derived colorectal cohort comprised 360 patients, including 65 source-defined positive and 295 MSS cases, represented by 192,312 pre-extracted patches. Patch embeddings were aggregated using mean, max, and mean-plus-max pooling, and downstream classifiers were evaluated under patient-level cross-validation with calibration, threshold, decision-curve, clinicopathological, source-provenance, and patch-count sensitivity analyses. **Results:** In the primary random patient-level evaluation, logistic regression with mean-plus-max pooling achieved a mean ROC-AUC of 0.915 and PR-AUC of 0.809. Fully nested Platt calibration yielded pooled out-of-sample ROC-AUC 0.913, PR-AUC 0.796, Brier score 0.073, calibration slope 1.037, and calibration-in-the-large 0.023. Within the internally cross-validated mean-plus-max framework, an exploratory sensitivity ≥ 0.95 operating point yielded sensitivity 0.969, specificity 0.393, and negative predictive value 0.983. Provenance reconstruction identified source-partition-dependent patch-count structure. In a source-TRAIN to source-TEST holdout, mean-plus-max ROC-AUC decreased to 0.788, while patch count alone was near chance in source-TEST (ROC-AUC 0.517) and explicit patch-count addition did not improve discrimination. **Conclusions:** These findings support the methodological feasibility of fixed foundation-model embeddings for internal MSI-related pre-screening research, while showing that apparent performance is sensitive to source structure. Independent multicenter validation with clinically adjudicated MSI-H/dMMR endpoints remains necessary before clinical application.

## 1. Introduction

Microsatellite instability (MSI) is a molecular phenotype resulting from deficient DNA mismatch repair and the consequent accumulation of insertion and deletion errors within repetitive genomic regions [1,2]. In colorectal cancer, MSI is associated with a distinct hypermutated tumor biology and has important implications for prognostic assessment, therapeutic stratification, and response to immune checkpoint blockade [1,3]. Although MSI is defined at the molecular level, MSI-high tumors may also exhibit recognizable histomorphological and immune-related characteristics on routine hematoxylin and eosin (H&E)-stained sections, including prominent lymphocytic infiltration, mucinous differentiation, architectural heterogeneity, and variation in the tumor microenvironment [1,3,4]. This relationship between molecular status and tissue morphology makes MSI a particularly relevant target for computational pathology, in which routinely acquired histological images can be analyzed as high-dimensional biomedical data.

In current clinical practice, MSI or mismatch repair status is generally assessed using polymerase chain reaction-based microsatellite testing or immunohistochemistry for the mismatch repair proteins MLH1, MSH2, MSH6, and PMS2 [1,3]. These established approaches remain essential for definitive biomarker assessment but require testing beyond routine morphological examination. By contrast, H&E-stained slides are already generated as part of standard histopathological workflows and may contain image-level correlates of the underlying molecular phenotype. Computational analysis of these routinely available images has therefore been investigated as a potential pre-screening approach for identifying patients who may benefit from prioritized confirmatory testing [4,5]. Such methods should not be considered replacements for validated molecular or immunohistochemical assays, but rather as complementary strategies that may support prioritization within existing diagnostic pathways.

The digitization of histopathological slides has enabled artificial intelligence methods to extract clinically relevant information from whole-slide images. Landmark work by Kather et al. demonstrated that deep learning could predict MSI directly from H&E-stained histological images of gastrointestinal cancers, establishing an important proof of concept for image-based molecular biomarker inference [5]. Subsequent studies and reviews have expanded this evidence base and further demonstrated the potential of computational pathology for MSI prediction [4,6,7]. However, the published literature also shows substantial heterogeneity in cohort composition, class prevalence, image preprocessing, model architecture, validation design, reference standards, and clinical framing [4,6,7]. The central question has therefore progressed beyond whether MSI-associated information is present in histomorphology to how this information should be represented, validated, and interpreted to support reliable, transparently evaluated, and potentially transferable patient-level inference.

Methodological rigor is particularly important in whole-slide image analysis because individual patients may contribute multiple slides and thousands of correlated image patches. If these observations are partitioned without preserving patient-level independence, related samples may occur in both the training and evaluation sets, creating information leakage and producing optimistically biased performance estimates [8]. Moreover, discrimination alone does not fully characterize the potential relevance of a biomarker prediction model. Although the area under the receiver operating characteristic curve (ROC-AUC) summarizes the ability of a model to rank positive and negative cases, it does not establish whether predicted probabilities are appropriately calibrated, whether operating thresholds suitable for a proposed use can be identified, or whether model-guided action may offer net benefit relative to default strategies [9,10,11]. Evaluation should therefore extend to class-imbalance-sensitive metrics, uncertainty estimation, probability calibration, threshold-dependent performance, and decision-analytic assessment. Robustness to variation in the amount of available tissue information is also relevant because whole-slide images may differ substantially in tissue area and patch burden. Accordingly, an unresolved methodological need is a patient-level framework that combines strict leakage control with calibration, operating-point analysis, decision curve evaluation, and representation-robustness assessment.

Task-specific end-to-end deep-learning pipelines can require substantial computational resources, extensive model optimization, and careful management of cohort-dependent effects [6,7,8]. Pathology foundation models provide a modular alternative by learning transferable representations from large and heterogeneous histopathology collections [12,13,14]. In an embedding-based workflow, image patches are processed using a fixed pretrained encoder, after which the resulting feature vectors can be aggregated into slide- or patient-level representations for downstream prediction. This separation of representation learning from task-specific classification reduces the need for repeated end-to-end retraining and allows the effects of representation design, classifier selection, calibration, and robustness to be examined within a common feature space. Within such a framework, deterministic mean and max pooling provide a transparent means of testing whether pretrained embeddings retain sufficient globally distributed and locally salient information without introducing an additional trainable attention mechanism. The resulting representations can then be evaluated using comparatively simple downstream models, improving analytical traceability and facilitating representation-level interpretation. Nevertheless, latent embedding dimensions should not be assumed to correspond directly to specific microscopic structures, and an embedding-based workflow does not eliminate the computational burden of patch extraction and foundation-model inference.

Against this background, the present study developed and internally evaluated a calibration-aware, leakage-safe, and decision-support-oriented framework for patient-level prediction of the source-defined MSIMUT category using a publicly available TCGA-linked colorectal cancer patch archive derived from routine H&E-stained whole-slide images [15,16]. The source repository defines MSIMUT as microsatellite unstable or highly mutated; accordingly, this category was treated as the positive analytical endpoint and was not assumed to be equivalent to a uniformly assay-confirmed MSI-H/dMMR outcome. The verified cohort comprised 360 patients, and patch-level features were extracted using the fixed Phikon pathology foundation model [17]. Mean pooling, max pooling, and their concatenation were evaluated for constructing patient-level representations, and multiple downstream classifiers were compared under strict patient-level separation. Evaluation extended beyond conventional discrimination analysis by incorporating repeated patient-level validation, bootstrap-based uncertainty estimation, leakage-safe nested probability calibration, threshold-based screening assessment, decision curve analysis, pooling-strategy comparison, patch-burden sensitivity analysis, and representation-level interpretability. The objective was to determine whether fixed Phikon-derived histopathology embeddings could support informative patient-level prediction that remained stable across alternative internal patient-level partitions, without end-to-end retraining, while explicitly characterizing probability behavior, operating-point trade-offs, potential decision-analytic relevance, and representation robustness.

## 2. Materials and Methods

### 2.1. Study Design, Data Source, and Cohort Assembly

**Figure 1:**
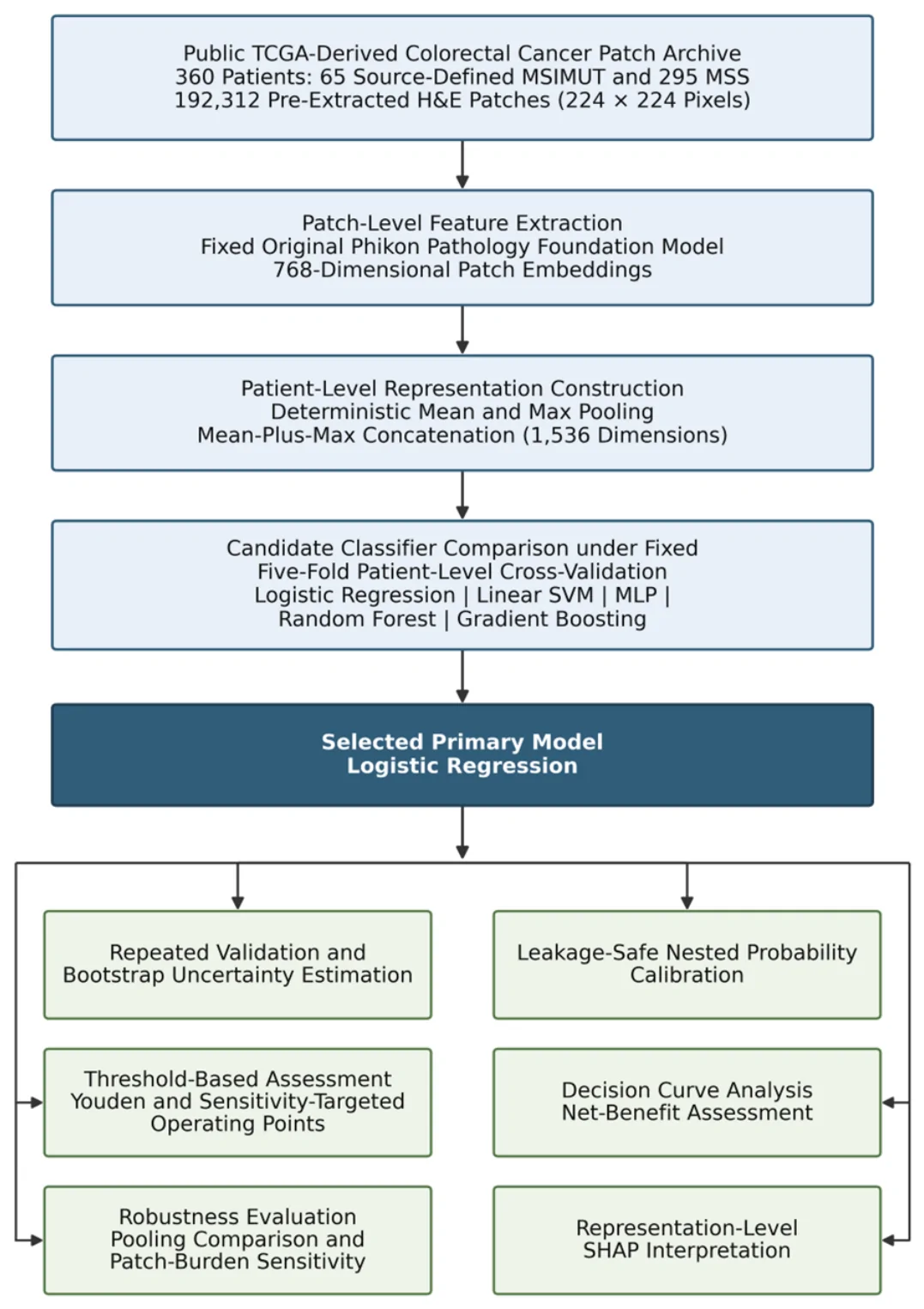
Overview of the patient-level MSI prediction workflow. Whole-slide-derived histopathology patches and their source-defined MSIMUT/MSS labels were obtained from a publicly available TCGA-derived colorectal cancer dataset. The pre-extracted image patches were encoded using the original Phikon pathology foundation model and aggregated into patient-level representations. Candidate classifiers were evaluated under patient-level validation, and the selected model subsequently underwent uncertainty estimation, leakage-safe nested probability calibration, threshold-based assessment, decision curve analysis, robustness evaluation, and representation-level interpretation.

This retrospective computational pathology study investigated patient-level prediction of microsatellite instability-related status using a publicly available colorectal histopathology patch dataset derived from hematoxylin and eosin (H&E)-stained whole-slide images. The patient was prespecified as the fundamental analytical unit because individual cases could contribute multiple correlated image-derived observations. Maintaining patient-level independence was intended to preserve the clinical interpretation of the prediction task and to reduce the risk of information leakage and optimistically biased performance estimates [6,8]. Accordingly, patient-level separation was maintained throughout representation construction and model evaluation. The overall study workflow is summarized in Figure 1.

Whole-slide-derived colorectal histopathology patches were obtained from a publicly available colorectal-only JPEG derivative [18] of the FFPE image dataset originally published by Kather et al. [16] and generated from TCGA diagnostic slides. The distributed archive was organized into source-defined MSIMUT and microsatellite-stable (MSS) categories. TCGA patient identifiers encoded in the patch filenames were extracted programmatically to reconstruct patient-level groups and to preserve the association between each patch and its source case. The source-provided category labels were used as the reference outcome for supervised prediction; no local relabeling or additional pathological annotation was performed. Patient-level groups were retained when the archive contained image patches with an extractable TCGA case identifier and a corresponding source-defined category label. No new participants were recruited, no intervention or specimen collection was undertaken, and no identifiable patient information was analyzed.

Following reconstruction and verification of the patient-level groups, the final analytical cohort comprised 360 patients, including 65 patients in the source-defined MSIMUT category and 295 patients in the MSS category. The positive category therefore represented 18.1% of the cohort, resulting in a class-imbalanced prediction problem. The approaches used to preserve class distribution during validation and to evaluate performance under class imbalance are described in the subsequent methodological sections. The verified cohort was held fixed throughout all representation construction, model evaluation, and robustness analyses, ensuring consistency between cohort definition and reported performance [6,8]. No independent external validation cohort was available for the present study. Accordingly, all discrimination, calibration, threshold, decision-analytic, subgroup, and sensitivity analyses should be interpreted as internal evaluations of the TCGA-derived cohort. Additional internal sensitivity analyses were designed to examine potential site- and clinicopathological confounding within this cohort, but they were not considered substitutes for independent multicenter validation across different institutions, staining protocols, scanners, specimen-processing workflows, or patient populations. The principal cohort characteristics and the prespecified analytical design are summarized in Table 1.

### 2.2. Source Patch Dataset and Patch-Level Feature Extraction

Image analysis was based on a publicly available colorectal histopathology patch collection published by Kather et al. [16] and subsequently distributed as a colorectal-only JPEG archive [18]. The original image resource was derived from formalin-fixed, paraffin-embedded diagnostic whole-slide images in The Cancer Genome Atlas. According to the source documentation, tumor-containing regions were detected automatically, resampled into 224 × 224-pixel patches at a spatial resolution of 0.5 µm per pixel, and color-normalized using the Macenko method [16]. The locally used archive contained 192,312 JPEG patches, comprising 75,039 patches in the source-defined MSIMUT directory and 117,273 patches in the microsatellite-stable (MSS) directory [18]. Provenance reconstruction showed that the upstream resource had been distributed in predefined TRAIN and TEST partitions with class-specific patch-count structures shaped by upstream dataset construction. The source TRAIN partition contained 46,704 MSIMUT and 46,704 MSS patches; according to the source documentation, this patch-level balance reflected random reduction of the more numerous MSS training patches [16]. The reconstructed source TEST partition contained 28,335 MSIMUT and 70,569 MSS patches. These counts refer to image files reconstructed from the available source filename manifest and matched to the 360-patient analytical cohort. At the patient level, these partitions comprised 260 TRAIN patients (39 MSIMUT and 221 MSS) and 100 TEST patients (26 MSIMUT and 74 MSS), with no patient overlap. Because the pooled derivative archive combined patients originating from these differently structured source partitions, absolute patient-level patch burden could encode upstream dataset-construction characteristics in addition to the amount of available tissue. This possibility was therefore examined explicitly through source-partition-specific patch-count analyses and a reconstructed source-holdout sensitivity analysis described below. The archive was supplied as a colorectal-only image collection, and no additional filtering by TCGA project, tissue-source site, or other source-related metadata was applied before patient-level representation construction. Because the analysis used pre-extracted patches rather than reprocessing the original whole-slide images, the original tiling stride and overlap configuration could not be determined from the available source documentation.

The source repository defines MSIMUT as microsatellite unstable or highly mutated [16]. In the present analysis, this source-defined category constituted the positive class, whereas MSS constituted the negative class. TCGA case identifiers embedded in the patch filenames were extracted programmatically and used to associate each patch with its source patient and to establish the patient-level identifier framework subsequently used for linkage to TCGA/GDC metadata. This procedure reconstructed 360 non-overlapping patient groups, comprising 65 patients in the source-defined positive category and 295 patients in the MSS category. No patient identifier occurred in both categories. Each patch was retained only as an intermediate image-derived observation, whereas the patient was preserved as the indivisible analytical unit throughout downstream dataset partitioning, model development, and performance evaluation; patches originating from the same patient were never distributed across training and test subsets. No additional image augmentation or histology-specific stain normalization was applied during feature extraction because the distributed JPEG patches had already undergone source-level color normalization.

**Figure 2:**
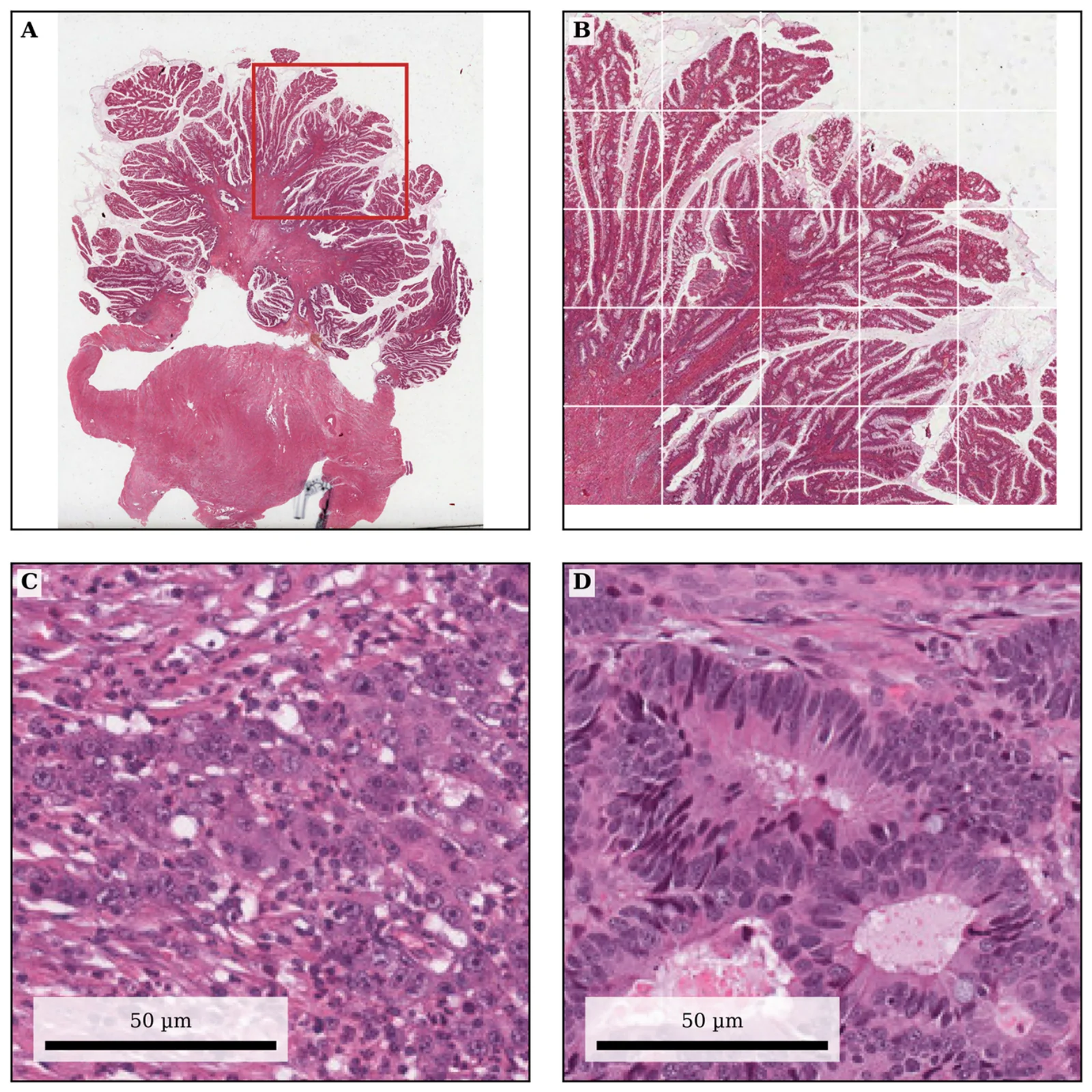
Whole-slide-derived image patches and patch-level feature extraction. (**A**) Low-resolution overview of a representative H&E-stained diagnostic whole-slide image corresponding to TCGA-CA-5796-01Z-00-DX1 (source WSI identifier: TCGA-CA-5796-01Z-00-DX1.88141789-4240-4ab7-8db1-e4cb7ee1ebda). (**B**) Schematic illustration of patch-based processing based on a selected tissue region from the same whole-slide image; the red rectangle in panel A indicates the region illustrated in panel (**B**). The displayed grid is schematic and does not reconstruct the original patch-extraction coordinates, tiling stride, or overlap configuration. (**C**) Representative 224 × 224-pixel patch from the source-defined MSIMUT category (TCGA case: TCGA-CM-4743). (**D**) Representative 224 × 224-pixel patch from the MSS category (TCGA case: TCGA-AY-A8YK). Panels (**C**,**D**) are provided solely to illustrate the appearance and dimensions of the model inputs and are not intended as comparative or class-discriminative histomorphological examples. Each patch was processed using the fixed original Phikon encoder to generate a 768-dimensional embedding. Scale bars = 50 µm.

Patch-level features were extracted using the original Phikon pathology foundation model, loaded through the Transformers interface using the model identifier owkin/phikon [17]. Each patch was opened in RGB format and processed using the corresponding AutoImageProcessor. The encoder was maintained in evaluation mode, and feature extraction was performed without gradient computation, task-specific fine-tuning, or end-to-end retraining. For each patch, the classification-token representation from the final hidden state was retained as a fixed 768-dimensional latent histopathology embedding. The embeddings were converted to 32-bit floating-point arrays and saved separately for each patient. Implementation used the corresponding AutoImageProcessor and AutoModel interfaces, with the encoder operated in evaluation mode under torch.no_grad() during patch-level inference. These fixed patch-level embeddings constituted the inputs for the deterministic patient-level aggregation procedures described in Section 2.3. The reconstructed patient identifiers were retained unchanged across downstream analyses and provided the linkage key for the source-related metadata analyses described separately below. The use of fixed pretrained pathology embeddings as transferable representations is consistent with broader general-purpose foundation-model approaches in computational pathology [19]. The overall patch-level feature-extraction workflow and representative model inputs are shown in Figure 2.

#### Reconstruction of the Original Source Partition and Source-Holdout Evaluation

To evaluate performance under a provenance-defined partition that was independent of the random patient-level folds used in the primary analysis, the original TRAIN/TEST partition of the upstream Kather dataset was reconstructed from the Zenodo source filename manifest. TCGA patient barcodes were extracted from the source filenames and mapped to the corresponding patients in the 360-patient analytical cohort. The reconstruction matched all 360 patients and all 192,312 patches, with no patient occurring in both source partitions. The reconstructed source-TRAIN partition comprised 260 patients, including 39 source-defined MSIMUT and 221 MSS cases, whereas the source-TEST partition comprised 100 patients, including 26 MSIMUT and 74 MSS cases. At the patch level, source TRAIN contained 46,704 MSIMUT and 46,704 MSS patches, while source TEST contained 28,335 MSIMUT and 70,569 MSS patches. The balanced patch counts in source TRAIN are consistent with the source-documented random reduction of the larger MSS training-patch set, whereas source TEST retained an unbalanced class-specific patch-count structure [16]. Counts reported here refer to image files reconstructed from the available source filename manifest and matched to the analytical cohort. These reconstructed source partitions were not used to define the primary five-fold cross-validation and were examined as a provenance-based sensitivity analysis.

A source-holdout evaluation was then performed by fitting the prespecified logistic-regression pipeline exclusively on the 260 source-TRAIN patients and applying the fitted pipeline once to the untouched 100 source-TEST patients. Mean-only, max-only, and mean-plus-max patient-level representations were evaluated separately using the same model configuration as in the primary analysis. Median imputation and feature standardization were estimated exclusively from source-TRAIN data and were subsequently applied unchanged to source-TEST data. Classifier parameters were not re-optimized for this analysis, and the source-TEST cohort was not used for preprocessing, model fitting, representation tuning, probability calibration, or operating-threshold selection.

Source-holdout discrimination was quantified using ROC-AUC and PR-AUC, and probabilistic accuracy was additionally summarized using the Brier score. Uncertainty in source-TEST ROC-AUC was estimated using 5000 class-stratified patient-level bootstrap resamples. Pairwise differences in ROC-AUC between the evaluated representations were estimated using paired bootstrap resampling, with identical resampled source-TEST patients used for both members of each comparison; models were not refitted during bootstrap resampling. As an additional provenance sensitivity analysis, raw patient patch count was treated as a univariable discrimination score, and its ROC-AUC and PR-AUC were calculated separately within the reconstructed source-TRAIN and source-TEST partitions. To determine whether absolute patch burden provided transportable information beyond the image-derived representations in the source-holdout setting, log1p-transformed patient patch count was also appended as one additional feature to the mean-only, max-only, and mean-plus-max patient-level representations. A count-only logistic-regression model using the same log1p-transformed patch-count feature was evaluated in parallel. For each augmented representation, the same prespecified logistic-regression pipeline was fitted exclusively on source-TRAIN and applied once to source-TEST. Paired bootstrap resampling of the source-TEST patients was then used to estimate the change in ROC-AUC produced by adding explicit patch count to each corresponding image representation. Models were not refitted during bootstrap resampling. Because both partitions originated from the same upstream TCGA-derived resource rather than from independent institutions or acquisition domains, this analysis was interpreted as a source-structured internal holdout and not as independent external validation.

### 2.3. Patient-Level Representation Construction

For each patient i, let $z_{ij}\in R^{768}$ denote the embedding of the j-th patch, where $j=1,\ldots ,n_{i}$ and $n_{i}$ is the number of patches associated with that patient. The complete set of patch embeddings for patient i was represented by the matrix $Z_{i}\in R^{n_{i}\times 768}$, with $z_{ij}$ corresponding to its j-th row. Because the number of available patches varied among patients, these variable-sized embedding matrices could not be used directly by the downstream classifiers, which required one fixed-length feature vector per patient. Each patient’s embedding matrix was therefore aggregated independently using deterministic and label-free pooling operations.

Two complementary pooling operations were applied along the patch dimension. Mean pooling generated a 768-dimensional vector by averaging each embedding dimension across all patches belonging to the patient:

(1) $$\mu_{i}=\frac{1}{n_{i}}\sum_{j=1}^{n_{i}}z_{\mathrm{ij}}$$

Max pooling generated a second 768-dimensional vector by retaining the largest observed activation in each embedding dimension:

(2) $$m_{i,k}=\max_{1\leq j\leq n_{i}}z_{\mathrm{ij},k},\;k=1,\ldots ,768$$

Mean pooling summarized the overall latent activation profile across the patient’s available tissue patches, whereas max pooling retained the strongest patch-level activation observed within each latent dimension. The two operations were treated as complementary because target-associated information could plausibly be broadly distributed across the available patches or concentrated within a smaller subset.

The principal patient-level representation was constructed by concatenating the mean-pooled vector followed by the max-pooled vector:

(3) $$x_{i}=\left[\mu_{i}\;∥\;m_{i}\right]\in R^{1536}$$

where ∥ denotes vector concatenation. This procedure produced a 360×1536 patient-level feature matrix, with one row per patient. The first 768 dimensions corresponded to mean-pooled features, and the remaining 768 dimensions corresponded to max-pooled features. Mean-only and max-only matrices, each of size 360×768, were also retained for the pooling-strategy comparison described in Section 2.7. The mean-plus-max representation was used as the principal input for candidate classifier comparison and subsequent model evaluation. The notebook implementation confirms that patient-specific embedding matrices were pooled using mean(axis = 0) and max(axis = 0) and concatenated in mean-then-max order to generate the 360 × 1536 matrix.

No global feature scaling was applied during representation construction. For scale-sensitive classifiers, feature standardization was performed within the model pipeline using parameters estimated exclusively from the training portion of each validation fold, as detailed in Section 2.4. Because the representation dimension exceeded the number of patients, with *p* = 1536 and *n* = 360, model-specific complexity control and strict patient-level validation were applied at the classifier stage to limit optimistic performance estimation.

The aggregation procedure contained no trainable parameters and did not use class-label information to determine patch contributions. Unlike attention-based multiple-instance learning approaches, in which instance contributions are estimated through learned attention weights, no patch-level attention scores or learned instance weights were estimated in the present framework [20,21,22]. This deterministic design provided a reproducible bridge between variable-sized patient patch collections and fixed-length patient-level representations while allowing globally averaged and maximal embedding information to be evaluated both jointly and separately.

### 2.4. Candidate Classifiers and Patient-Level Validation

Five downstream classifiers were evaluated using the same 1536-dimensional mean-plus-max patient representation: logistic regression, a linear support vector machine (linear SVM), a multilayer perceptron (MLP), random forest, and gradient boosting. These algorithms were selected to span linear, neural-network-based, and tree-based modelling approaches with different levels of complexity. Candidate comparison was treated as an empirical model-selection step because the relative performance of linear and nonlinear downstream learners may depend on the encoder, aggregation strategy, cohort, and validation design [23,24,25].

Fixed model configurations were applied consistently across all validation folds. Logistic regression was implemented using median imputation and feature standardization within each training fold, the lbfgs solver, L2 regularization with C = 1.0, a convergence tolerance of 1 × 10^−4^, a maximum of 5000 iterations, balanced class weights, and a random seed of 42. The Linear SVM used median imputation, feature standardization, a linear kernel, probabilistic output estimation, balanced class weights, and the same random seed. Random forest was configured with 500 decision trees, a random seed of 42, and parallel processing across the available CPU cores. Gradient boosting was preceded by median imputation and was fitted using a random seed of 42. The MLP comprised two hidden layers with 128 and 64 neurons, respectively, rectified linear unit activation, the Adam optimizer, an L2 penalty parameter of 1 × 10^−4^, an initial learning rate of 1 × 10^−3^, a maximum of 300 iterations, and a random seed of 42. Median imputation and feature standardization were also included in the MLP pipeline. Parameters not explicitly specified were retained at the defaults of the recorded software environment.

Preprocessing operations were incorporated within the corresponding model pipelines. Consequently, median values and standardization parameters were estimated exclusively from the training patients in each fold and were subsequently applied to the held-out test patients. No preprocessing parameter was estimated from the complete cohort before cross-validation. Standardization was applied to logistic regression, the Linear SVM, and the MLP, whereas the tree-based models were fitted without feature scaling. No feature selection, dimensionality reduction, or data-driven hyperparameter search was performed during candidate classifier comparison.

Primary internal validation was performed using stratified five-fold cross-validation with shuffling and a fixed random seed of 42. Patient-level identifiers, rather than image patches, were assigned to the folds. Each fold contained 288 training patients and 72 held-out test patients. The class distribution was preserved across folds, with each test fold containing 13 patients from the source-defined positive category and 59 MSS patients. Every patient therefore contributed to the held-out test set exactly once, and no patient or patch derived from that patient could occur simultaneously in the training and test partitions of the same fold.

For each classifier and fold, the probability assigned to the positive category was obtained for every held-out patient. Threshold-independent performance was assessed using the area under the receiver operating characteristic curve (ROC-AUC) and the area under the precision–recall curve (PR-AUC). Because the cohort was class imbalanced, PR-AUC was considered an important complementary measure to ROC-AUC. At the conventional probability threshold of 0.50, accuracy, balanced accuracy, precision, sensitivity, F1 score, Matthews correlation coefficient, and confusion-matrix counts were calculated. The Brier score was additionally reported to quantify the overall accuracy of the predicted probabilities.

Candidate-model performance was summarized as the mean and standard deviation across the five held-out folds. The classifier with the highest mean ROC-AUC, with PR-AUC considered as a secondary discrimination criterion, was designated as the primary model and carried forward to the repeated-validation, uncertainty, calibration, threshold, and decision-analytic evaluations described in the subsequent sections.

To quantify potential optimism arising from selection of the primary analytical pipeline within the same cohort, an additional nested pipeline-selection sensitivity analysis was performed. The original five outer patient-level test folds were kept completely untouched. Within each corresponding outer-training set, five-fold stratified inner cross-validation was used to compare the same five prespecified analytical pipelines and fixed model configurations used in the primary classifier comparison. Candidate selection was based on mean inner ROC-AUC, with mean inner PR-AUC used as a prespecified tie-breaker. The selected pipeline was then refitted on the complete outer-training set and evaluated once on the corresponding untouched outer-test fold. No outer-test observation contributed to pipeline selection. Before performing the nested analysis, the exact candidate configurations were required to reproduce the original fixed-fold classifier results within a predefined numerical tolerance. This nested analysis was intended to quantify pipeline-selection sensitivity and potential selection optimism rather than to establish intrinsic superiority of an algorithm family.

An additional sensitivity analysis was performed to examine whether the primary logistic-regression result depended strongly on the specified regularization strength or class weighting. Using the original fixed five patient-level folds, L2-regularized logistic regression was evaluated at C values of 0.01, 0.1, 1, 10, and 100 with both balanced and unweighted class objectives. The solver (lbfgs), convergence tolerance (1 × 10^−4^), maximum number of iterations (5000), random seed (42), median imputation, and within-training-fold standardization were otherwise held fixed. This analysis was prespecified as a sensitivity analysis and was not used to retrospectively optimize or replace the primary C = 1 balanced logistic-regression pipeline. Convergence warnings were recorded for each evaluated configuration.

### 2.5. Repeated Validation, Bootstrap Uncertainty Estimation, and Calibration

To assess whether the performance of the selected primary logistic regression model depended on a single cross-validation partition, the complete model pipeline was additionally evaluated using repeated stratified five-fold patient-level cross-validation. The five-fold procedure was repeated 10 times using a fixed random seed of 42, yielding 50 held-out evaluations. In every split, 288 patients were used for model training, and 72 patients were reserved for testing. Stratification preserved the source-defined positive/MSS class distribution, and all preprocessing and model-fitting operations were performed using only the training portion of each split. Each patient appeared in a held-out test fold once per repeat and therefore received 10 out-of-sample probability estimates generated under different repeated partitions. Fold-level performance metrics were first averaged across the five folds within each repeat, after which the mean and standard deviation were calculated across the 10 repeat-level estimates. This two-stage summarization avoided treating the 50-fold-level results as statistically independent observations.

For patient-level uncertainty estimation, the 10 out-of-sample probabilities generated for each patient were averaged to obtain one patient-specific probability estimate. Point estimates of ROC-AUC, PR-AUC, accuracy, balanced accuracy, precision, sensitivity, F1 score, Matthews correlation coefficient, and Brier score were calculated from these patient-averaged probabilities. Threshold-dependent metrics at this stage used the default probability threshold of 0.50. A nonparametric bootstrap analysis was then performed using a NumPy random-number generator initialized with a fixed seed of 42. The 360 patient-level outcome–probability pairs were resampled with replacement, with each bootstrap sample containing 360 observations. Samples containing only one outcome class were discarded because discrimination metrics could not be calculated, and resampling continued until 2000 valid bootstrap samples had been obtained. Performance metrics were recalculated for every valid resample, and two-sided 95% confidence intervals were defined by the 2.5th and 97.5th percentiles of the corresponding empirical bootstrap distributions [26]. The prediction model was not refitted during this procedure; the bootstrap analysis therefore quantified uncertainty in the performance estimates derived from the fixed patient-averaged out-of-sample predictions.

Probability calibration was assessed using a fully nested procedure within each of the original five outer patient-level folds. For each outer fold, five-fold inner cross-validation was performed using only the corresponding outer-training patients to generate out-of-fold base-model decision scores. Three candidate probability strategies—uncalibrated probabilities, Platt scaling, and isotonic regression—were compared using the Brier score [27] calculated exclusively from cross-fitted outer-training predictions, consistent with standard supervised probability-calibration approaches [28,29,30]. The calibration strategy with the lowest inner cross-validated Brier score was selected without access to the outer-test outcomes. The selected calibration mapping was then fitted using the outer-training out-of-fold scores and applied once to the untouched outer-test base-model scores. Thus, no outer-test observation was used for calibration-method selection or calibrator fitting.

Platt scaling was implemented as an unweighted logistic mapping of the common base-model decision scores, whereas isotonic regression was implemented as an unweighted monotonic nonparametric mapping. A same-score audit was additionally performed to verify preservation of ROC ranking under Platt scaling. Because Platt scaling is strictly monotonic, its fold-specific ROC-AUC was required to equal the ROC-AUC of the corresponding uncalibrated outer-test scores. Isotonic regression was evaluated separately because its piecewise-constant mapping can introduce ties and therefore can alter rank-based discrimination metrics.

Calibration was characterized using the Brier score, a joint calibration intercept and slope estimated from logistic recalibration, and calibration-in-the-large (CITL). CITL was treated separately from the jointly estimated calibration intercept; ideal calibration corresponds to a joint calibration intercept of 0, a calibration slope of 1, and a CITL of 0. CITL was estimated as the intercept from an intercept-only logistic calibration model with the logit-transformed predicted probability entered as an offset, corresponding to a calibration slope fixed at 1. The nested-selected out-of-sample probabilities were carried forward to the threshold and decision-curve analyses.

### 2.6. Threshold-Based Screening and Decision Curve Analysis

Threshold-based analyses were repeated using the nested-selected Platt-calibrated out-of-sample probabilities generated by the procedure described in Section 2.5. For each evaluated threshold, patients with predicted probabilities greater than or equal to the threshold were classified as positive, whereas those with probabilities below the threshold were classified as negative. Operating-point performance was quantified using accuracy, balanced accuracy, sensitivity, specificity, positive predictive value, negative predictive value, F1 score, Matthews correlation coefficient, and the numbers of true-positive, true-negative, false-positive, and false-negative classifications.

The primary balanced operating point was identified using the Youden index:

(4) J=Sensitivity+Specificity−1

The observed probability threshold maximizing the Youden index was designated as the exploratory Youden threshold. To examine screening-oriented operating conditions, additional thresholds targeting sensitivities of at least 0.90 and 0.95 were evaluated. Candidate thresholds consisted of the unique nested-Platt-calibrated out-of-sample probabilities and were examined in descending order. For each sensitivity target, the highest probability threshold satisfying the prespecified requirement was selected, thereby retaining the greatest achievable specificity among the observed thresholds meeting that target. Because these operating points were derived and evaluated using the same internally generated out-of-sample probability set, they were interpreted as exploratory cohort-specific thresholds rather than externally validated clinical cutoffs.

Uncertainty in threshold-dependent performance was quantified using patient-level stratified bootstrap resampling. For each fixed operating threshold, the patient-level outcome–probability pairs were resampled with replacement, and the corresponding sensitivity, specificity, predictive values, classification metrics, and confusion-matrix-derived quantities were recalculated. Two-sided 95% bootstrap confidence intervals were defined by the 2.5th and 97.5th percentiles of the resulting empirical distributions. Threshold selection itself was not repeated within each bootstrap sample; these intervals therefore quantify uncertainty conditional on the internally derived operating thresholds.

Decision curve analysis was repeated using the nested-selected Platt-calibrated probabilities to assess potential decision-analytic value within a clinically focused low-threshold range [10,11]. Threshold probabilities from 0.01 to 0.30 were evaluated at increments of 0.005. At each threshold probability, model net benefit was calculated as:

(5) $${\mathrm{NB}}_{\mathrm{model}}\left(p_{t}\right)=\frac{\mathrm{TP}}{N}-\frac{\mathrm{FP}}{N}\left(\frac{p_{t}}{1-p_{t}}\right)$$

where TP and FP denote the numbers of true-positive and false-positive classifications, respectively, *N* denotes the total number of patients, and $p_{t}$ denotes the threshold probability. The false-positive term was weighted by the odds associated with the selected threshold probability, thereby incorporating the relative consequences of unnecessary confirmatory assessment and missed source-defined positive cases.

The calibrated model was compared with test-all and test-none reference strategies. For the test-all strategy, in which every patient was prioritized for confirmatory assessment, net benefit was calculated as:

(6) $${\mathrm{NB}}_{\mathrm{all}}\left(p_{t}\right)=\pi -\left(1-\pi \right)\left(\frac{p_{t}}{1-p_{t}}\right)$$

where π denotes the observed source-defined positive-class prevalence. For the test-none strategy, in which no patient was prioritized for confirmatory assessment, net benefit was defined as:

(7) $${NB}_{none}\left(p_{t}\right)=0$$

At each threshold probability, model net benefit was compared with the better of the two default strategies. Uncertainty in incremental net benefit was assessed using paired patient-level stratified bootstrap resampling, thereby preserving direct comparison between the model and the corresponding default strategy within each resample. Two-sided 95% bootstrap intervals were obtained from the empirical distribution of the paired net-benefit difference. The number of confirmatory assessments performed or avoided per 100 patients and the number of source-defined positive cases missed per 100 patients were additionally summarized to provide an interpretable description of the internal testing-burden trade-off.

Because calibration, operating-threshold derivation, and decision-curve evaluation were performed within the same retrospective TCGA-derived cohort, these analyses were considered exploratory internal assessments. They do not establish prospective clinical utility, cost-effectiveness, safety of rule-out decisions, or transportability to other clinical settings. Independent multicenter validation with prespecified calibration and operating thresholds would be required before the model could inform confirmatory testing or patient-care decisions.

For application to a previously unseen patient, the intended inference pathway would require a single frozen version of the complete analytical pipeline. Histopathology patches would first be encoded using the same fixed Phikon feature extractor and aggregated into the prespecified mean-plus-max patient representation. The imputation and standardization parameters learned from the development data would then be applied without refitting, followed by the frozen logistic-regression model to generate a base decision score. A prespecified Platt calibration mapping, fitted only from development data, would transform this score into a calibrated probability. That probability would then be compared with a prespecified operating threshold chosen according to the intended clinical use; patients at or above the threshold would be prioritized for confirmatory MSI-related assessment, whereas those below the threshold would not be considered model-positive. The present cross-validation analysis was designed for internal performance estimation and therefore did not generate or validate a deployment-ready single final model, calibrator, or clinical threshold. Before prospective use, these components would need to be refitted or frozen using the complete development set according to a prespecified protocol and then evaluated without modification in an independent external cohort.

### 2.7. Pooling Comparison, Patch-Burden and Patch-Count Sensitivity, and Interpretability

To determine whether patient-level predictive information was primarily retained in averaged or maximal embedding activations, three pooling representations were compared: mean-only pooling, max-only pooling, and their concatenated mean-plus-max representation. Mean-only and max-only pooling each produced a 768-dimensional feature vector per patient, whereas mean-plus-max pooling produced the principal 1536-dimensional representation described in Section 2.3. Each representation was evaluated using the same fixed five-fold patient-level partitions and the same primary logistic regression pipeline described in Section 2.4. All preprocessing and model-fitting operations were performed exclusively within the training portion of each fold, and balanced class weights were applied consistently across the three representations. No additional feature selection, dimensionality reduction, calibration, or hyperparameter optimization was performed for this comparison. Fold-level ROC-AUC, PR-AUC, accuracy, balanced accuracy, sensitivity, specificity, precision, negative predictive value, F1 score, Matthews correlation coefficient, and Brier score were calculated, with threshold-dependent metrics evaluated at the default probability threshold of 0.50. Performance was summarized as the mean and standard deviation across the five folds. Because the classifier configuration and patient partitions were held constant, differences in performance reflected changes in the pooling representation rather than changes in the downstream modeling procedure.

Patch burden sensitivity analysis was conducted to assess whether the performance of the principal mean-plus-max representation depended on the availability of the complete patch collection for each patient. Patch embeddings were randomly subsampled without replacement at fractions of 25%, 50%, 75%, and 100% of each patient’s available patches. For patient *i* and subsampling fraction *f*, the number of retained patches was defined as:

(8) $$n_{i,f}=\max\left(1,\left\lceil f\cdot n_{i}\right\rceil \right)$$

where $n_{i}$ denotes the total number of available patches for patient i, and $\left\lceil \cdot \right\rceil$ denotes the ceiling function. Mean-pooled and max-pooled vectors were recalculated from the retained patches and concatenated to reconstruct a 1536-dimensional patient representation. Subsampling was repeated 10 times for each fraction using fixed and reproducible random seeds. At the 100% reference condition, all available patches were retained; consequently, the reconstructed representations were identical across repetitions.

Each reconstructed dataset was evaluated using the same fixed five-fold patient-level partitions and the same primary logistic regression pipeline used in the main analysis. All preprocessing and classifier-fitting steps were confined to the corresponding training fold. For each patch fraction and repetition, performance was first averaged across the five folds. The mean and standard deviation were then calculated across the 10 repeat-level estimates. This two-stage procedure avoided treating the 50-fold–repeat evaluations within each patch fraction as statistically independent. The analysis assessed robustness to reduced patch availability within the existing patch archive and was not intended to reproduce variability arising from alternative tissue sampling, slide preparation, scanning systems, or external clinical sites.

To distinguish robustness to proportional reductions in patch availability from potential confounding by absolute patch count, an additional fixed-N sensitivity analysis was performed. Patients were randomly subsampled without replacement to fixed patch counts of N = 6, 25, 50, 100, and 200, with 10 independent repetitions at each N. For a given fixed-N condition, only patients with at least N available patches were eligible. The original patient-level fold assignments were retained after restriction to the eligible cohort. Mean-only, max-only, and mean-plus-max representations were reconstructed from the sampled patches and evaluated using the same logistic-regression pipeline as in the primary analysis. To separate the effect of fixed patch number from changes in cohort composition at higher N values, an all-patch comparator was recalculated using exactly the same eligible patients and the same restricted fold assignments for each N.

Patch-count dependence was additionally examined in two complementary analyses. First, class-specific patch-count distributions and a patch-count-only prediction model were evaluated to quantify the extent to which patch burden itself was associated with the source-defined outcome. Second, because max pooling may increase with the number of opportunities to observe extreme latent activations, each of the 768 max-pooled embedding dimensions was residualized with respect to log1p-transformed patient patch count within each outer-training fold only. Both linear and quadratic patch-count adjustment specifications were evaluated. The relationship estimated from the outer-training patients was then applied unchanged to the corresponding held-out patients. The resulting residualized max representation was evaluated alone and after concatenation with the original mean-pooled representation using the same primary logistic-regression pipeline. This analysis was designed as a sensitivity analysis of patch-count-associated representation variation and was not used to replace or retrospectively optimize the primary model. As an additional audit of the residualization procedure, out-of-fold patch-count decoding was performed to test whether multivariable patch-count information remained recoverable from the raw or residualized max-pooled representations. Using the same five patient-level outer folds, ridge regression models were trained to predict log1p-transformed patient patch count from the raw max-pooled representation, the linearly residualized max-pooled representation, and the quadratically residualized max-pooled representation. For the residualized analyses, the patch-count adjustment was fitted only within the corresponding outer-training fold before decoding models were trained. The resulting out-of-fold predictions were evaluated using R^2^, Spearman correlation, and mean absolute error. This analysis was used only to assess residual patch-count information in the representations and was not used for outcome prediction or model selection.

Representation-level interpretability was assessed using SHapley Additive exPlanations generated with a linear SHAP explainer. The analysis used the principal 1536-dimensional mean-plus-max representation and followed the same five fixed patient-level folds used for primary model evaluation. Within each fold, the preprocessing steps and primary logistic regression configuration described in Section 2.4 were fitted exclusively using the 288 outer training patients. The transformed outer-training matrix was subsequently provided to the linear explainer as the candidate background dataset. Because the number of training patients exceeded the masker’s default maximum background size, the SHAP Independent masker selected 100 training patients without replacement using its deterministic default random state of 0. SHAP values were then calculated only for patients in the corresponding held-out outer test fold. This procedure produced one out-of-sample SHAP vector for each of the 360 patients and prevented held-out patients from contributing to preprocessing, classifier fitting, or selection of the background distribution used to explain their predictions.

The Independent masker generated interventional feature attributions for the fitted linear classifier; correlations among the latent embedding dimensions were not explicitly incorporated into the attribution procedure. The first 768 dimensions of the representation were labeled as mean-pooled features, and the remaining 768 dimensions were labeled as max-pooled features. Global feature importance was defined as the mean absolute SHAP value across all out-of-sample patients. Importance was summarized separately for the mean-pooled and max-pooled halves of the representation. The 20 dimensions with the highest global mean absolute SHAP values were additionally examined to characterize the relative representation of mean-pooled and max-pooled components among the most influential latent dimensions. SHAP values were used solely for post-hoc interpretation and did not influence representation construction, feature selection, classifier selection, model fitting, calibration, or performance estimation.

Because the explained variables were latent foundation-model embedding dimensions rather than directly labeled histopathological attributes, the SHAP analysis was interpreted strictly at the representation level. A high SHAP importance value indicated greater reliance of the linear classifier on a particular pooled embedding dimension but did not identify a specific patch, tissue region, cell type, or recognizable microscopic structure. The analysis therefore did not provide spatial localization, direct morphological attribution, mechanistic biological interpretation, or evidence of causal relationships.

### 2.8. Clinicopathological and Site-Related Sensitivity Analyses

TCGA case identifiers reconstructed from the image archive were linked to publicly available GDC metadata to investigate whether clinicopathological or site-related factors could account for part of the observed predictive signal. All 360 cases were matched to GDC records and originated from TCGA-COAD or TCGA-READ. Available variables included age, AJCC pathologic stage, tissue or organ of origin, primary diagnosis, project identifier, and tissue-source-site (TSS) code. Sex/gender and a uniform clinical molecular MSI-test result were not included in the analyzable metadata extract used for the present sensitivity analyses; these variables therefore could not be incorporated into the sensitivity models. Separately, GDC-derived MSIsensor2 annotations were retrieved for a secondary concordance and endpoint-sensitivity analysis; these bioinformatics-derived molecular annotations were not treated as equivalent to a clinically adjudicated PCR-, immunohistochemistry-, or sequencing-confirmed MSI-H/dMMR reference standard.

Associations between the source-defined outcome and available clinicopathological variables were evaluated using the Mann–Whitney U test for age and permutation-based chi-square tests for categorical variables. To quantify the extent to which these variables alone could discriminate the source-defined classes, a clinical-only logistic-regression model was evaluated under the same fixed five patient-level folds. A second sensitivity model combined the 1536-dimensional image representation with age, project identifier, broad pathologic stage, tissue or organ of origin, and primary diagnosis. Numeric variables were imputed and standardized within each training fold, and categorical variables were imputed and one-hot encoded using training data only.

Potential site effects were further investigated using TSS code. A TSS-only model was evaluated under the fixed folds, and an additional TSS-group-isolated validation used grouped cross-validation so that all patients from a given TSS were confined entirely to either the training or test portion of a fold. These analyses were intended to assess sensitivity to clinicopathological and source-site structure within the available TCGA cohort and were not interpreted as external validation.

### 2.9. Software, Reproducibility, and Reporting Standards

The computational workflow was implemented in Python-based Google Colaboratory environments running on Linux platforms. Patch-level embedding extraction was performed in a CUDA-enabled runtime using an NVIDIA Tesla T4 GPU. The embedding-extraction notebook recorded Python 3.12.12 and approximately 14.56 GB of available GPU memory; however, the corresponding CUDA and cuDNN versions were not retained. The subsequent patient-level modeling, statistical evaluation, and final reproducibility audit were conducted using Python 3.12.13. The final verification environment included NumPy 2.0.2, pandas 2.2.2, scikit-learn 1.6.1, SciPy 1.16.3, statsmodels 0.14.6, Matplotlib 3.10.0, SHAP 0.52.0, PyTorch 2.11.0, torchvision 0.26.0, Transformers 5.13.1, Hugging Face Hub 1.23.0, Pillow 11.3.0, and openpyxl 3.1.5. Because package-level versions were not retained for every earlier computational run, these values characterize the final verification environment and should not be interpreted as a complete historical reconstruction of the initial embedding-extraction environment.

Patch-level feature extraction was implemented using PyTorch and the Transformers library. The original Phikon pathology foundation model was loaded using the repository identifier owkin/phikon and was used exclusively as a fixed feature extractor. The encoder was operated in evaluation mode under torch.no_grad(), and no end-to-end retraining or task-specific fine-tuning was performed. A commit-specific model revision was not explicitly pinned or recorded during the original embedding-generation run, and the exact repository snapshot used for embedding generation could not be reconstructed retrospectively. We therefore report the verified repository identifier rather than assigning an unverified revision number. To preserve downstream reproducibility, the exact patient-specific patch embeddings generated in the original run were retained and publicly archived together with the pooled representation matrices, cross-validation splits, random seeds, analysis configurations, out-of-sample predictions, and analysis code. These archived embedding arrays constitute the fixed analytical inputs used for all downstream analyses reported in this study.

A single verified patient-level dataset state was maintained throughout the analytical workflow. The retained data objects comprised 360 patients, including 65 source-defined positive cases and 295 MSS cases. The mean-pooled and max-pooled representation matrices each had dimensions of 360 × 768, and the concatenated mean-plus-max representation matrix had dimensions of 360 × 1536. All values in the final patient-level representation matrices were finite. Patient identifiers, source-defined labels, patient ordering, patch counts, representation matrices, and split definitions were retained consistently across classifier comparison, primary and repeated validation, bootstrap uncertainty estimation, calibration, threshold analysis, decision curve analysis, pooling comparison, patch burden sensitivity analysis, and representation-level interpretability.

The integrity of the fixed five-fold validation structure was verified programmatically. Each fold contained 288 training patients and 72 held-out patients, with no overlap between the training and test subsets. The union of the training and test subsets covered all 360 patients in every fold, and each patient appeared in a held-out test set exactly once across the five fixed partitions. The same fold definitions were reused whenever direct comparisons among classifiers, pooling strategies, calibration procedures, or robustness conditions were required, preventing variation in patient partitioning from being conflated with variation in the evaluated analytical method.

Randomized procedures were controlled using fixed and documented random seeds. A principal seed of 42 was used for Python, NumPy, PyTorch, and scikit-learn operations where applicable, including patient-level fold generation, repeated cross-validation, bootstrap resampling, classifier initialization, and patch subsampling. Method-specific deterministic defaults, including the SHAP background-sampling procedure described in Section 2.7, were documented separately. Explicit cuDNN deterministic backend settings were not recorded for the embedding-extraction run. Therefore, although the encoder was used exclusively in inference mode and no stochastic model training was performed, exact bitwise reproduction of the patch-level embeddings across different GPU, CUDA, cuDNN, or library configurations cannot be guaranteed. Downstream reproducibility was instead supported by preserving the generated embedding arrays as fixed analytical inputs.

Intermediate and final computational artifacts were saved programmatically to provide an auditable analysis record. Retained materials included the verified patient manifest, patient-order files, fixed split definitions, patch-level embedding arrays, mean-pooled, max-pooled, and mean-plus-max representation matrices, fold-level and patient-level prediction files, classifier-comparison results, repeated-validation summaries, bootstrap outputs, calibration outputs, threshold-performance tables, confusion-matrix counts, decision-curve outputs, pooling-comparison files, patch-subsampling summaries, and out-of-sample SHAP values and importance rankings. Outputs were stored in comma-separated value, spreadsheet, NumPy, JavaScript Object Notation, and image formats, as appropriate. Runtime and analysis-configuration records were additionally retained in machine-readable formats. As an integrity measure, SHA-256 checksums were generated for 15 critical data and result files, including the verified representation matrices, labels, patient ordering, split manifest, calibration outputs, threshold results, pooling and patch-burden summaries, and out-of-sample SHAP values.

The reporting of this retrospective prediction-model study was guided by the TRIPOD+AI statement and the 2024 update of the Checklist for Artificial Intelligence in Medical Imaging [31,32]. Particular emphasis was placed on transparent cohort and outcome definition, preservation of the patient as the unit of analysis, leakage-safe validation, explicit description of preprocessing and representation construction, reporting of class imbalance, discrimination and calibration assessment, uncertainty estimation, threshold-dependent operating characteristics, decision curve analysis, robustness evaluation, and separation of representation-level attribution from spatial or biological interpretation. These guidelines were used as reporting frameworks rather than as evidence of complete checklist compliance, prospective clinical validation, regulatory evaluation, or deployment readiness. Data and code availability arrangements are reported separately in the corresponding availability statements.

## 3. Results

### 3.1. Cohort, Patch-Level Data, and Patient-Level Representation

The final verified cohort comprised 360 patients. Of these, 65 patients belonged to the source-defined positive category designated as MSIMUT in the original repository, whereas 295 patients belonged to the MSS category. The source-defined positive class therefore accounted for 18.1% of the cohort, resulting in an imbalanced binary outcome distribution. The same cohort and source-defined class assignments were used in all subsequent analyses.

The colorectal histopathology archive contained 192,312 JPEG patches linked to the included patients. Patch availability varied substantially across patients, with a mean of 534.2 patches per patient (standard deviation, 699.9), a median of 291.5 patches (interquartile range, 119.8–592.3), and a range of 6 to 4305 patches. The patches served as intermediate inputs for feature extraction, whereas model development and performance evaluation were conducted at the patient level.

Each retained patch was encoded as a 768-dimensional vector using the fixed Phikon pathology foundation model. The number of patch embeddings available for an individual patient corresponded to that patient’s patch count. All available embeddings for each patient were used to construct the primary representation, without imposing a common patch-count requirement across patients.

Deterministic pooling converted each patient’s variable-length set of patch embeddings into fixed-length representations. Mean pooling produced a 360 × 768 matrix, and max pooling produced a second 360 × 768 matrix. Concatenation of the mean-pooled and max-pooled vectors yielded the primary 360 × 1536 analytical matrix, corresponding to one 1536-dimensional feature vector for each of the 360 patients.

An overview of the cohort and analytical representation is provided in Table 1. Detailed cohort composition and patch-count characteristics are reported in Supplementary Table S1.

### 3.2. Candidate Classifier Comparison and Primary Model Selection

Five candidate classifiers were evaluated using the same 1536-dimensional mean-plus-max patient-level representation and the same fixed five-fold patient-level partitions. Logistic regression achieved the numerically highest mean discrimination, with a ROC-AUC of 0.915 ± 0.035 and a PR-AUC of 0.809 ± 0.035. At the default probability threshold of 0.50, it yielded an accuracy of 0.908, balanced accuracy of 0.836, precision of 0.760, sensitivity of 0.723, specificity of 0.949, F1 score of 0.737, Matthews correlation coefficient of 0.685, and Brier score of 0.076.

Linear support vector machine and MLP produced discrimination estimates close to those of logistic regression. Their mean ROC-AUC values were both approximately 0.911, and their mean PR-AUC values were 0.798 and 0.803, respectively. Linear support vector machine achieved the highest precision among the evaluated implementations, at 0.799, together with a specificity of 0.966, but its sensitivity was lower at 0.569. MLP achieved a sensitivity of 0.646 and specificity of 0.946, with a Brier score of 0.084 compared with 0.077 for Linear support vector machine and 0.076 for logistic regression.

The two tree-based implementations showed less favorable positive-class recovery at the default threshold. Random forest achieved a mean ROC-AUC of 0.886 and PR-AUC of 0.696, but its sensitivity was 0.092 and its specificity was 1.000. Gradient boosting achieved a mean ROC-AUC of 0.826 and PR-AUC of 0.609, with sensitivity of 0.292 and specificity of 0.969. Consequently, their overall accuracies of 0.836 and 0.847 were driven largely by correct classification of the majority MSS class and did not reflect balanced recovery of both outcome groups.

These threshold-dependent findings apply specifically to the fixed model implementations and the default threshold of 0.50 used in the comparison. Logistic regression and linear support vector machine incorporated balanced class weights, whereas the evaluated MLP, random forest, and gradient boosting implementations used unweighted objectives. No classifier-specific threshold optimization, post-hoc calibration, hyperparameter search, or harmonized imbalance treatment was performed at this stage. The results therefore should not be interpreted as demonstrating intrinsic inferiority of the corresponding algorithm families.

According to the predefined ranking procedure, which prioritized mean ROC-AUC and subsequently mean PR-AUC, logistic regression ranked first among the five candidate implementations. It additionally achieved the lowest Brier score and the highest Matthews correlation coefficient. Differences in discrimination among logistic regression, linear support vector machine, and MLP were numerically modest, and no formal pairwise statistical superiority test was performed. Nevertheless, logistic regression provided the most favorable combined profile of discrimination, positive-class recovery, classification balance, probability accuracy, and model parsimony within the prespecified comparison and was therefore selected as the primary model for repeated validation, uncertainty estimation, calibration, threshold analysis, decision curve analysis, robustness assessment, and representation-level interpretability.

The nested pipeline-selection sensitivity analysis reproduced the original fixed-fold results for all five candidate pipelines before nested selection was undertaken. Logistic regression was subsequently selected within the outer-training data in all five outer folds. The nested-selected pipeline therefore achieved the same mean outer-fold ROC-AUC of 0.915 ± 0.035 and PR-AUC of 0.809 ± 0.035 as the fixed logistic-regression reference evaluated on the same untouched outer-test folds. The corresponding pooled out-of-sample ROC-AUC and PR-AUC were 0.915 and 0.802, respectively, for both approaches. Accordingly, the observed paired differences between the nested-selected and fixed logistic-regression predictions were 0.000 for both ROC-AUC and PR-AUC. These findings indicate that, within this internal sensitivity analysis, the original selection of logistic regression was reproduced independently within every outer-training set; however, the comparison remains internal to the same cohort and should not be interpreted as evidence of intrinsic algorithm-family superiority. Detailed fold-level results are provided in Supplementary Table S14.

Regularization-sensitivity analysis showed that discrimination was relatively stable across the more strongly regularized C = 0.01–1 range. With balanced class weights, mean ROC-AUC values were 0.923, 0.917, and 0.915 at C = 0.01, 0.1, and 1, respectively, with corresponding mean PR-AUC values of 0.822, 0.812, and 0.809. Performance decreased with weaker regularization, with mean ROC-AUC values of 0.903 and 0.892 at C = 10 and 100, respectively. At C = 1, removing class weighting produced the same mean ROC-AUC of 0.915 and a similar PR-AUC of 0.805 compared with 0.809 for the balanced configuration. No convergence warnings occurred across any of the evaluated regularization or class-weight settings. Because this was a sensitivity analysis rather than a hyperparameter-selection procedure, the original balanced C = 1 configuration was retained as the primary model. Complete regularization and class-weight results are provided in Supplementary Table S15.

The principal classifier-comparison results are summarized in Table 2. Detailed classifier metrics and confusion-matrix-derived summaries are provided in Supplementary Table S2 and Table S3, respectively.

### 3.3. Primary Model Stability, Uncertainty, and Calibration

Across the five fixed patient-level folds, the selected logistic regression model achieved ROC-AUC values ranging from 0.863 to 0.957 and PR-AUC values ranging from 0.751 to 0.838. Fold-level classification and probability-performance results are reported in Supplementary Tables S4 and S5.

Repeated stratified five-fold patient-level cross-validation, comprising 10 repeats and 50 held-out evaluations, yielded a mean ROC-AUC of 0.911 ± 0.007 and a mean PR-AUC of 0.797 ± 0.020. At the default probability threshold of 0.50, repeat-level mean accuracy was 0.901 ± 0.008, balanced accuracy was 0.826 ± 0.013, precision was 0.739 ± 0.031, sensitivity was 0.709 ± 0.025, F1 score was 0.718 ± 0.023, Matthews correlation coefficient was 0.662 ± 0.028, and Brier score was 0.080 ± 0.006. The small between-repeat standard deviations and the proximity of these estimates to those obtained from the original fixed five-fold analysis supported the internal stability of the primary model across alternative patient-level partitions.

Averaging the 10 held-out probability estimates available for each patient produced one patient-specific out-of-sample probability for bootstrap uncertainty estimation. Based on these patient-averaged probabilities, ROC-AUC was 0.914, PR-AUC was 0.806, accuracy was 0.900, balanced accuracy was 0.831, precision was 0.723, sensitivity was 0.723, F1 score was 0.723, Matthews correlation coefficient was 0.662, and Brier score was 0.071. The corresponding classification comprised 47 true-positive, 277 true-negative, 18 false-positive, and 18 false-negative predictions at the default threshold of 0.50. The equality of precision, sensitivity, and F1 score resulted from the equal numbers of false-positive and false-negative classifications.

The 2000-resample bootstrap analysis produced 95% confidence intervals of 0.859–0.956 for ROC-AUC and 0.708–0.889 for PR-AUC. The corresponding intervals were 0.867–0.931 for accuracy, 0.772–0.886 for balanced accuracy, 0.607–0.829 for precision, 0.611–0.828 for sensitivity, 0.623–0.809 for F1 score, 0.552–0.764 for Matthews correlation coefficient, and 0.051–0.095 for Brier score. Because these estimates were obtained by resampling the patient-averaged internally held-out predictions from the same 360-patient cohort without refitting the model, they quantify uncertainty in internal performance and do not represent validation in an independent external cohort.

Calibration was re-evaluated using the fully nested procedure described in Section 2.5. Platt scaling was selected in all five outer folds on the basis of cross-fitted Brier scores calculated exclusively within the corresponding outer-training data. For the pooled uncalibrated outer-test predictions, ROC-AUC was 0.915, PR-AUC was 0.802, and the Brier score was 0.076. The joint calibration intercept was −0.407 and the calibration slope was 0.403, whereas calibration-in-the-large (CITL) was 0.040. Because the primary logistic-regression model was fitted using a balanced-class objective and evaluated within the present development cohort, the probability-scale characteristics of the uncalibrated predictions, including the estimated calibration slope and intercept, were interpreted descriptively and were not attributed to a single source of calibration error. After nested Platt calibration, pooled out-of-sample ROC-AUC was 0.913, PR-AUC was 0.796, and the Brier score improved to 0.073. The corresponding joint calibration intercept was 0.057, the calibration slope was 1.037, and CITL was 0.023. These results indicate substantially improved probability calibration after leakage-safe Platt scaling while preserving strong discrimination.

A same-score audit confirmed that Platt scaling produced exactly the same ROC-AUC as the corresponding uncalibrated base-model scores within each of the five outer folds, consistent with the strictly monotonic nature of the logistic mapping. The small difference between pooled uncalibrated and pooled Platt ROC-AUC values arose because fold-specific calibrated probabilities were subsequently combined across outer folds. Isotonic regression yielded a pooled Brier score of 0.076 and calibration slope of 0.687; because its piecewise-constant mapping introduced tied scores, it did not preserve fold-specific ROC-AUC exactly. The nested-selected Platt probabilities were therefore carried forward to the threshold-based screening and decision-curve analyses. Detailed repeated-validation, bootstrap, and nested calibration results are provided in Table 3 and Supplementary Tables S6 and S7.

### 3.4. Threshold-Based Screening Performance and Decision Curve Results

Threshold-based analyses were repeated using the nested-selected Platt-calibrated out-of-sample probabilities from the internally cross-validated mean-plus-max primary pipeline described in Section 2.5. At the exploratory Youden operating point, the selected probability threshold was 0.2474. This threshold yielded an accuracy of 0.878, balanced accuracy of 0.865, sensitivity of 0.846, specificity of 0.885, positive predictive value of 0.618, negative predictive value of 0.963, F1 score of 0.714, and Matthews correlation coefficient of 0.652. The corresponding confusion matrix comprised 55 true-positive, 261 true-negative, 34 false-positive, and 10 false-negative classifications. Overall, 89 patients were assigned to the positive-prediction group and 271 to the negative-prediction group.

At the screening-oriented operating point targeting sensitivity of at least 0.90, the highest-specificity threshold satisfying the target was 0.1327. Sensitivity was 0.908 and specificity was 0.786, with a positive predictive value of 0.484, negative predictive value of 0.975, accuracy of 0.808, balanced accuracy of 0.847, F1 score of 0.631, and Matthews correlation coefficient of 0.564. Of the 65 source-defined positive patients, 59 were classified as positive and 6 were missed. Among the 295 MSS patients, 63 were classified as positive. Overall, 122 patients were assigned to the positive-prediction group and 238 to the negative-prediction group.

For the operating point targeting sensitivity of at least 0.95, the selected threshold was 0.0219. Sensitivity reached 0.969 and negative predictive value reached 0.983, whereas specificity decreased to 0.393 and positive predictive value to 0.260. This threshold yielded an accuracy of 0.497, balanced accuracy of 0.681, F1 score of 0.410, and Matthews correlation coefficient of 0.297. It identified 63 of the 65 source-defined positive patients, while 179 MSS patients were classified as positive. Overall, 242 patients were assigned to the positive-prediction group and 118 to the negative-prediction group.

These operating points illustrate the expected trade-off between positive-class detection and confirmatory-testing burden. Relative to testing all patients, the sensitivity ≥ 0.90 operating point would have reduced the number of confirmatory assessments by approximately 66.1 per 100 patients while missing approximately 1.67 source-defined positive patients per 100. At the sensitivity ≥ 0.95 operating point, approximately 32.8 confirmatory assessments per 100 patients would have been avoided, while approximately 0.56 source-defined positive patients per 100 would have been missed. These calculations quantify an internal triage trade-off only and do not establish that confirmatory testing can safely be withheld. Because the operating points were derived and evaluated using the same internally generated out-of-sample probability set from the random patient-level mean-plus-max framework, they should be regarded as exploratory cohort-specific thresholds rather than source-holdout-tested, patch-count-neutral, or clinically validated cutoffs.

Decision curve analysis was repeated using the nested-Platt probabilities over a clinically focused threshold-probability range of 0.01 to 0.30. Model net benefit was compared with both test-all and test-none strategies, and uncertainty was assessed using patient-level stratified bootstrap resampling. The observed model net benefit exceeded both default strategies at several low thresholds; however, the lower 2.5th percentile of the paired bootstrap difference relative to the better default strategy remained above zero only from approximately 0.055 through 0.30. The exploratory sensitivity ≥0.95 operating threshold of 0.0219 was therefore below the range in which bootstrap-supported superiority over the better default strategy was observed.

**Figure 3:**
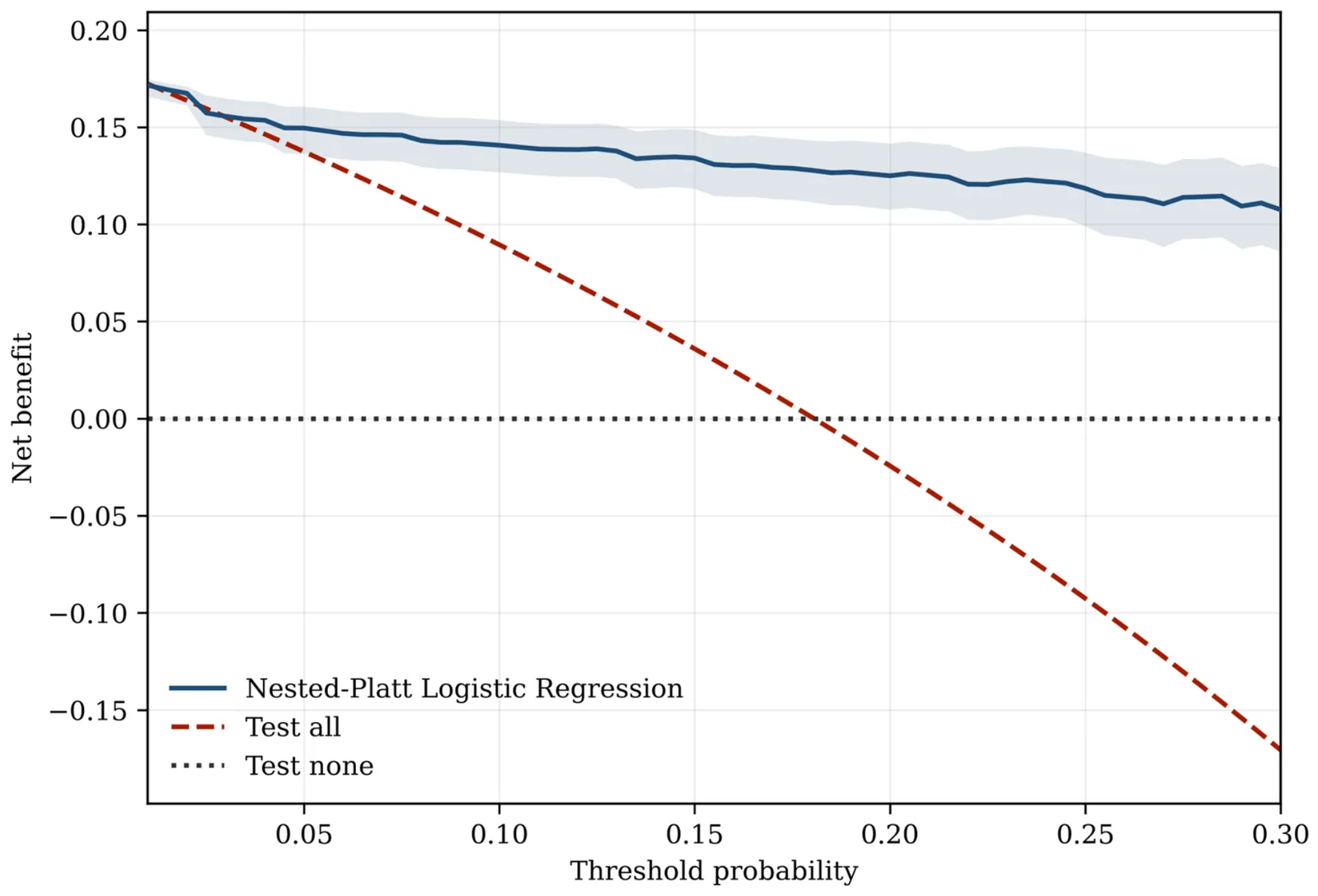
Decision curve analysis of the nested-Platt-calibrated primary logistic regression model. Net benefit is shown for the calibrated model, the test-all strategy, and the test-none strategy across threshold probabilities ranging from 0.01 to 0.30. The lower 2.5th percentile of the paired bootstrap difference between the model and the better default strategy remained above zero from approximately 0.055 through 0.30; the exploratory sensitivity ≥ 0.95 operating threshold of 0.0219 was below this bootstrap-supported range. These results represent an exploratory internal decision-analytic assessment and do not establish prospective clinical utility, cost-effectiveness, safety of rule-out decisions, source-holdout performance, or externally validated operating thresholds.

For example, at a threshold probability of 0.10, model net benefit was 0.141 compared with 0.090 for the test-all strategy, and the paired bootstrap difference relative to the better default strategy was 0.051 with a 95% interval of 0.037 to 0.064. At a threshold probability of 0.15, model net benefit was 0.134 compared with 0.036 for test-all, with a corresponding difference of 0.098 and a 95% interval of 0.082 to 0.113. These results indicate potential internal decision-analytic value within the evaluated low-threshold range. However, they do not establish prospective clinical utility, cost-effectiveness, safety of rule-out decisions, or transportability to other clinical settings. Independent multicenter validation would be required before any operating threshold could inform confirmatory testing or patient-care decisions. Threshold-specific performance is summarized in Table 4, the decision curve is presented in Figure 3, and detailed numerical outputs are provided in Supplementary Tables S8 and S9.

### 3.5. Pooling Strategy, Patch-Burden and Patch-Count Sensitivity, and Representation-Level Interpretability

Pooling strategies showed different predictive performance profiles under the original fixed five-fold patient-level cross-validation framework. Under the same fixed five-fold patient-level partitions and the same primary logistic regression pipeline, mean-plus-max pooling achieved the numerically most favorable overall performance, with a mean ROC-AUC of 0.915 ± 0.035, PR-AUC of 0.809 ± 0.035, Matthews correlation coefficient of 0.685, and Brier score of 0.076. Max-only pooling yielded a similar mean ROC-AUC of 0.912 ± 0.036 but a lower PR-AUC of 0.767 ± 0.062, Matthews correlation coefficient of 0.618, and a higher Brier score of 0.085. Mean-only pooling showed lower performance, with a mean ROC-AUC of 0.820 ± 0.046, PR-AUC of 0.592 ± 0.076, Matthews correlation coefficient of 0.440, and Brier score of 0.141. Although no formal pairwise statistical comparison was performed at this stage, the mean-plus-max representation showed the most favorable numerical profile across the prespecified metrics within the original fixed-fold analysis and was therefore retained as the primary representation for the main analytical pipeline.

Patch-subsampling analysis showed limited variation in performance when patient-level representations were reconstructed using 25%, 50%, 75%, or 100% of the available patches. After performance was first averaged across the five folds within each repetition and then summarized across the 10 repeat-level estimates, the 25% condition yielded a ROC-AUC of 0.915 ± 0.005, PR-AUC of 0.794 ± 0.015, and Matthews correlation coefficient of 0.655 ± 0.030. The corresponding values were 0.919 ± 0.004, 0.807 ± 0.008, and 0.691 ± 0.017 at the 50% condition and 0.915 ± 0.003, 0.804 ± 0.008, and 0.684 ± 0.014 at the 75% condition. When all patches were retained, ROC-AUC was 0.915, PR-AUC was 0.809, and Matthews correlation coefficient was 0.685.

No monotonic improvement was observed as the retained patch fraction increased. The 50% condition produced the numerically highest ROC-AUC and Matthews correlation coefficient, whereas the 100% condition produced the highest PR-AUC. These differences were small and were not tested for statistical significance; they therefore do not indicate that patch subsampling improved predictive performance. At the 100% condition, all available patches were retained in every repetition, producing identical reconstructed representations and repeat-level standard deviations of zero by construction. The analysis supports robustness to reduced patch availability within the existing archive but does not reproduce variability associated with alternative tissue sampling, slide preparation, scanning systems, patch-generation procedures, or external clinical sites.

Absolute patch burden differed substantially between outcome groups. Source-defined MSIMUT patients had a mean of 1154.4 patches per patient (median, 854), compared with 397.5 patches per patient (median, 243) among MSS patients. Across the complete 360-patient cohort, patch count was associated with class assignment: the raw and log1p-transformed patch counts each yielded a univariable ROC-AUC of 0.819, and a patch-count-only model evaluated under the fixed patient-level folds achieved a mean ROC-AUC of 0.817 and PR-AUC of 0.536. These pooled-cohort findings demonstrated an association between patch burden and the source-defined outcome and motivated additional sensitivity analyses addressing both absolute patch count and source-partition structure.

When the number of sampled patches was fixed across eligible patients, discrimination was attenuated relative to the corresponding all-patch comparator. Across fixed-N values from 6 to 200, the mean ROC-AUC of the mean-plus-max representation ranged from approximately 0.810 to 0.854, whereas the corresponding all-patch baselines recalculated on the same eligible cohorts ranged from approximately 0.898 to 0.916. A similar attenuation was observed for max-only pooling. Because eligibility changed at N > 6, comparisons at each N were made only against the all-patch model evaluated in exactly the same eligible patient subset.

A complementary fold-contained patch-count-adjustment analysis produced a similar but more nuanced result. The median absolute Spearman correlation between patch count and the raw max-pooled embedding dimensions was 0.411 and decreased to 0.023 after linear residualization and 0.014 after quadratic residualization. The raw mean-plus-max model achieved a mean ROC-AUC of 0.915, whereas the mean-plus-residualized-max representations achieved ROC-AUCs of 0.898 and 0.894 under the linear and quadratic adjustments, respectively. Both adjusted combined representations remained more discriminative than the mean-only model (ROC-AUC, 0.820) within the original random patient-level folds. However, this residualization result should be interpreted together with the source-holdout analysis, in which max-containing representations did not show a transportable advantage over mean-only pooling. These findings indicate that absolute patch burden contributed to the predictive information carried by the raw max-pooled representation, while residualized random-fold performance alone is insufficient to establish source-independent incremental value of the max-pooled component.

To further assess whether multivariable patch-count information remained recoverable after residualization, an out-of-fold ridge-regression decoding audit was performed using log1p-transformed patient patch count as the target. Raw max-pooled embeddings strongly encoded patch count, with an out-of-fold R^2^ of 0.8742, Spearman ρ of 0.9199, and mean absolute error of 0.3467. After fold-contained linear residualization, patch-count decoding was no longer useful by R^2^ and mean absolute error (out-of-fold R^2^, −0.0123; mean absolute error, 0.9880), although a small inverse rank association remained (Spearman ρ, −0.1314; *p* = 0.0126). Quadratic residualization produced the same summary values in the stored decoding audit (out-of-fold R^2^, −0.0123; Spearman ρ, −0.1314; *p* = 0.0126; mean absolute error, 0.9880). These results extend the dimension-wise correlation audit by showing that fold-contained residualization substantially attenuated useful multivariable patch-count decoding from the max-pooled representation under this decoding analysis, although the residualized representations were not interpreted as completely independent of patch count. Detailed results are provided in Supplementary Tables S20 and S21.

In the fold-contained out-of-sample SHAP analysis, the mean absolute SHAP contribution averaged across the mean-pooled half of the representation was 0.0296, compared with 0.0377 for the max-pooled half. Among the 20 embedding dimensions with the highest global mean absolute SHAP values, 16 originated from the max-pooled component and four from the mean-pooled component. The highest-ranked individual dimension was max_246, with a mean absolute SHAP value of 0.1505. These findings indicate that the fitted linear classifier placed greater average reliance on latent dimensions derived from max pooling. Given the strong patch-count encoding observed in the raw max-pooled representation, highly ranked max-pooled SHAP dimensions may partly reflect patch-burden or source-preprocessing proxies rather than localized biological signal. Nevertheless, the more favorable predictive profile of the combined representation relative to max-only pooling within the random-fold analysis was consistent with an additional contribution from the mean-pooled component.

The greater contribution of max-pooled dimensions was compatible with reliance on strong latent activations present within a subset of the available patches, but it may also reflect the greater opportunity to observe extreme activations in patients with more patches. Because the embedding dimensions were not linked to specific tissue characteristics and the raw max-pooled representation strongly encoded patch count, this finding does not establish that the model detected spatially localized histopathological hotspots or any particular MSI-associated morphological feature. Specific hypotheses involving lymphocytic infiltration, mucinous morphology, or other recognizable tissue patterns therefore require separate spatially resolved investigation.

The SHAP results describe model reliance on latent embedding dimensions and should not be interpreted as identifying particular patches, tissue regions, cell types, microscopic structures, or biological mechanisms. In addition, because the interventional SHAP masker did not explicitly model correlations among the embedding dimensions, the reported attribution magnitudes may differ under correlation-aware explanation methods. The analysis therefore provides representation-level interpretability rather than spatial, morphological, mechanistic, or causal explanation.

**Figure 4:**
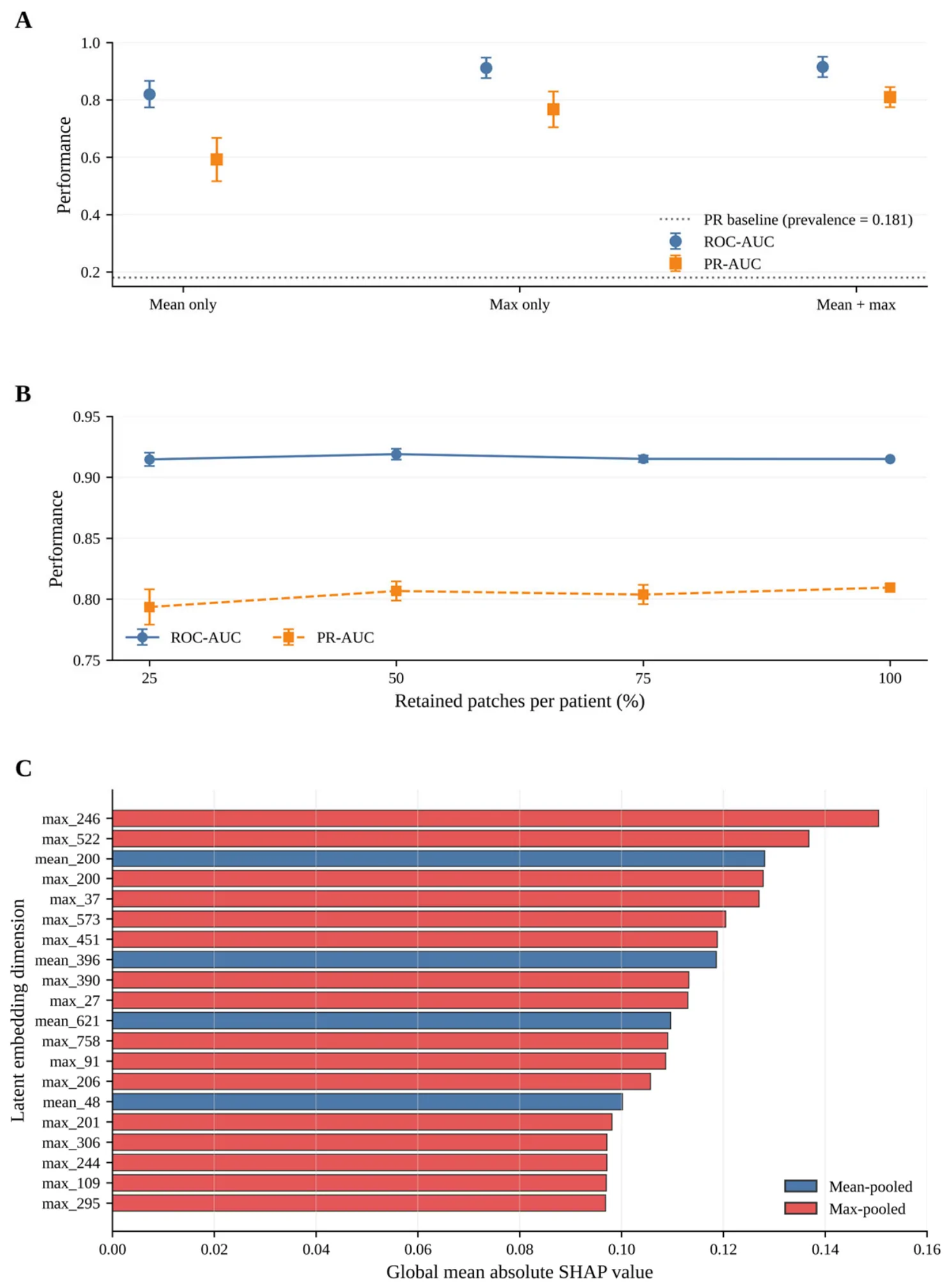
Pooling-strategy comparison, patch-burden robustness, and representation-level interpretability of the primary logistic regression model. (**A**) Performance of mean-only, max-only, and concatenated mean-plus-max patient-level representations under the same fixed five-fold patient-level cross-validation framework. Error bars for ROC-AUC and PR-AUC represent standard deviations across the five held-out folds. (**B**) ROC-AUC and PR-AUC obtained after retaining 25%, 50%, 75%, or 100% of each patient’s available patches. For the 25%, 50%, and 75% conditions, error bars represent standard deviations across 10 repeat-level estimates after performance was first averaged across the five folds within each repetition. At the 100% condition, all patches were retained and the reconstructed representations were identical across repetitions; the corresponding repeat-level standard deviations were therefore zero by construction. (**C**) Global mean absolute SHAP values for the 20 highest-ranked latent embedding dimensions in the fold-contained out-of-sample interpretability analysis. Sixteen of the top 20 dimensions originated from the max-pooled half and four from the mean-pooled half. The highest-ranked dimension was max_246, with a mean absolute SHAP value of 0.1505. SHAP contributions are interpreted at the representation level and are not directly mapped to specific histological structures or biological mechanisms; because raw max-pooled embeddings strongly encoded patch count, highly ranked max-pooled dimensions may also reflect patch-burden or source-preprocessing proxies.

Pooling results are summarized in Figure 4A and Supplementary Table S10, patch-burden results in Figure 4B and Supplementary Table S11, and representation-level SHAP results in Figure 4C and Supplementary Tables S12 and S13.

Because patch burden may itself reflect upstream dataset-construction or source-related characteristics, an additional provenance-based analysis was performed using the reconstructed original source partition to distinguish random-fold robustness from performance under a predefined source holdout.

### 3.6. Reconstructed Source-Partition Holdout Analysis

The original source-defined TRAIN/TEST partition of the upstream Kather dataset was successfully reconstructed for all 360 patients and all 192,312 analyzed patches, with no patient overlap between the two partitions. The reconstructed source-TRAIN set comprised 260 patients (39 source-defined MSIMUT and 221 MSS), whereas the source-TEST set comprised 100 patients (26 MSIMUT and 74 MSS). At the patch level, source-TRAIN contained 46,704 MSIMUT and 46,704 MSS patches, whereas source-TEST contained 28,335 MSIMUT and 70,569 MSS patches. These values refer to image files reconstructed from the available source filename manifest and matched to the analytical cohort. The source-documented random reduction of the larger MSS training-patch set provides a direct mechanism for the class-dependent patch-count structure observed in source-TRAIN. This partition therefore provided a provenance-defined holdout that was distinct from the random patient-level folds used in the primary cross-validation analysis.

When the prespecified logistic-regression pipeline was fitted exclusively on source-TRAIN and evaluated once on the untouched source-TEST cohort, the mean-only representation achieved a ROC-AUC of 0.794 (95% CI, 0.671–0.898), PR-AUC of 0.668 (95% CI, 0.511–0.843), and Brier score of 0.174 (95% CI, 0.111–0.243). Max-only pooling yielded a ROC-AUC of 0.766 (95% CI, 0.655–0.861), PR-AUC of 0.548 (95% CI, 0.408–0.716), and Brier score of 0.263 (95% CI, 0.195–0.335). The mean-plus-max representation achieved a ROC-AUC of 0.788 (95% CI, 0.679–0.881), PR-AUC of 0.593 (95% CI, 0.446–0.767), and Brier score of 0.259 (95% CI, 0.189–0.333). Thus, discrimination remained above chance under the reconstructed source holdout, but performance was attenuated relative to the original random-fold evaluation, and the numerical ordering of the pooling representations differed from that observed in the primary analysis.

In paired bootstrap comparisons on the same source-TEST patients, the ROC-AUC difference between mean-only and mean-plus-max pooling was 0.0057 (95% CI, −0.0764 to 0.0852), whereas the difference between mean-only and max-only pooling was 0.0281 (95% CI, −0.0759 to 0.1289). Both confidence intervals included zero, providing no clear evidence of a pairwise ROC-AUC difference among these representations in the source-holdout cohort. Accordingly, the numerically higher mean-only performance in source-TEST should not be interpreted as demonstrating superiority over the combined representation.

Patch-count discrimination was strongly dependent on the reconstructed source partition. Raw patient patch count yielded a ROC-AUC of 0.918 and PR-AUC of 0.815 within source-TRAIN but only a ROC-AUC of 0.517 and PR-AUC of 0.330 within source-TEST. The near-chance ROC-AUC in source-TEST contrasts with the stronger patch-count association observed in the pooled cohort and demonstrates that patch burden was not a stable discriminator across the predefined source partition.

Explicitly adding log1p-transformed patch count to the image-derived representations did not materially improve source-TEST discrimination. The count-only logistic-regression model yielded a ROC-AUC of 0.5169 and PR-AUC of 0.3301. Adding patch count changed ROC-AUC only minimally for mean-only pooling (0.7937 to 0.7957; paired ΔROC-AUC, 0.0021; 95% CI, −0.0218 to 0.0244), max-only pooling (0.7656 to 0.7640; Δ, −0.0016; 95% CI, −0.0078 to 0.0042), and mean-plus-max pooling (0.7879 to 0.7827; Δ, −0.0052; 95% CI, −0.0125 to 0.0000). Thus, explicit patch-count information did not provide a transportable improvement in the source-holdout setting.

Taken together, the source-holdout analysis indicates that part of the apparent performance under random patient-level partitioning was sensitive to upstream source structure and patch-burden-related preprocessing history. Mean-only performance changed less across validation schemes, from ROC-AUC 0.820 in the primary fixed-fold analysis to 0.794 in source-TEST, whereas mean-plus-max performance decreased from 0.915 to 0.788. In this dataset, the more source-robust internal discrimination therefore appeared closer to the 0.79–0.82 range than to the primary random-fold mean-plus-max estimate. At the same time, image-derived representations retained moderate discriminatory information in the untouched source-TEST cohort despite the loss of patch-count discrimination and the lack of improvement after explicit patch-count addition. Because both source partitions originated from the same upstream TCGA-derived resource, these findings represent a source-structured internal robustness analysis rather than independent external validation.

### 3.7. Clinicopathological and Site-Related Sensitivity Analyses

GDC linkage confirmed that the cohort contained both TCGA-COAD and TCGA-READ cases. Age, project identifier, broad pathologic stage, tissue or organ of origin, and primary diagnosis showed measurable associations with the source-defined outcome, indicating that clinicopathological structure was not independent of class assignment. TSS code also showed a non-negligible effect size, although its permutation-based association test did not reach the conventional 0.05 threshold.

The clinical-only model achieved a mean fold ROC-AUC of 0.770 and PR-AUC of 0.433, whereas the image-only model achieved 0.915 and 0.809, respectively. Combining the available clinical variables with the image representation yielded mean ROC-AUC of 0.916 and PR-AUC of 0.808. In paired bootstrap analysis, the difference between the image-plus-clinical and image-only models was 0.0002 for ROC-AUC (95% CI, −0.0025 to 0.0031) and 0.0016 for PR-AUC (95% CI, −0.0059 to 0.0098), providing no clear evidence of incremental discrimination from these covariates under the evaluated pipeline.

A TSS-only model showed limited discrimination, with a mean ROC-AUC of 0.586 and PR-AUC of 0.227. When all patients sharing a TSS were isolated entirely between training and test groups, mean ROC-AUC decreased to 0.867 ± 0.047 and mean PR-AUC to 0.714 ± 0.101. This attenuation indicates that source-site structure may contribute to the apparent predictive performance and should not be ignored; however, substantial discrimination remained under TSS-isolated internal validation. These internal sensitivity analyses reduce, but do not eliminate, concern regarding clinicopathological and site-related confounding. Detailed clinicopathological association, clinical-comparison, and TSS sensitivity results are provided in Supplementary Tables S16–S18.

## 4. Discussion

### 4.1. Principal Findings

This study developed a calibration-aware, patient-level framework for predicting the source-defined positive category from colorectal histopathology using fixed pathology foundation-model embeddings. Its principal contribution was not the introduction of another end-to-end image architecture, but the integration of leakage-safe patient-level modeling, uncertainty estimation, calibration assessment, threshold-dependent evaluation, decision curve analysis, representation testing, and interpretability within a single reproducible workflow. Within the internally evaluated cohort, this approach demonstrated that pretrained histopathology embeddings could support informative patient-level prediction without retraining the image encoder.

Logistic regression provided the most favorable combined profile among the five prespecified classifier implementations, achieving a primary ROC-AUC of 0.915. Its selection reflected the predefined ranking procedure, probability quality, positive-class recovery, and model parsimony rather than formal statistical superiority over Linear SVM or MLP, which showed similar discrimination. Repeated cross-validation and bootstrap analyses indicated that performance was not narrowly dependent on a single random patient-level partition. However, the reconstructed source-partition analysis provided a more stringent provenance-based test: when the prespecified logistic-regression pipeline was trained on the reconstructed source-TRAIN cohort and evaluated on the untouched source-TEST cohort, ROC-AUC decreased from 0.915 in the primary random-fold analysis to 0.788 for the mean-plus-max representation. By contrast, the mean-only representation changed from 0.820 in the primary fixed-fold analysis to 0.794 in source-TEST. This pattern indicates that the magnitude of discrimination observed under random patient-level cross-validation, especially the apparent increment associated with adding max pooling, was partly sensitive to upstream source structure. The more source-robust internal discrimination observed here was therefore closer to approximately 0.79–0.82 than to the primary random-fold mean-plus-max estimate. Nevertheless, above-chance discrimination was retained in the source-defined holdout, supporting the presence of predictive information within the fixed embedding representation while arguing against interpreting the primary cross-validation estimate as source-independent performance. This substantially attenuated source-holdout performance was therefore treated as a central interpretive limitation of the framework rather than as a secondary sensitivity finding.

The nested calibration analysis materially changed the interpretation of probability performance. When calibration-method selection was nested entirely within the outer-training data, Platt scaling was selected in all five folds. The uncalibrated predictions showed a calibration slope below 1 (0.403) with a CITL of 0.040. Because the primary logistic-regression model used a balanced-class objective and was evaluated within the present development cohort, these probability-scale characteristics were interpreted descriptively rather than attributed to a single source of calibration error. After leakage-safe nested Platt calibration, the calibration slope was 1.037 and CITL was 0.023, with the pooled Brier score improving from 0.076 to 0.073. The same-score audit further confirmed that Platt scaling preserved fold-specific ROC-AUC exactly, whereas isotonic regression could alter rank-based metrics through the introduction of tied probabilities. These results address the optimism inherent in the original post-hoc calibration comparison but remain internal estimates requiring external reassessment.

The clinicopathological sensitivity analyses also qualify interpretation of the image-derived signal. Age, broad pathologic stage, tissue or organ of origin, project membership, and primary diagnosis were associated with the source-defined outcome, confirming that clinically relevant covariate structure is present within the cohort. Nevertheless, the clinical-only model discriminated substantially less well than the image model, and addition of the audited clinical variables produced essentially no incremental improvement over image-only prediction. TSS-group-isolated validation produced lower performance than the original fixed-fold analysis but retained substantial discrimination. The reconstructed source-holdout analysis showed a further attenuation of the primary mean-plus-max ROC-AUC from 0.915 under random patient-level cross-validation to 0.788 when training and testing followed the original source partition. Considered together, these analyses indicate that clinicopathological, site-related, and broader source-provenance structure can influence apparent model performance. At the same time, the persistence of above-chance discrimination under both TSS-isolated and source-holdout evaluation suggests that these measured source effects do not fully account for the predictive signal. Residual confounding by unmeasured morphological, specimen-related, preprocessing-related, or center-specific technical factors nevertheless cannot be excluded.

Threshold analysis also showed that no single operating point was uniformly optimal. Lowering the threshold reduced missed source-defined positive cases but substantially increased the number of MSS patients prioritized for confirmatory assessment. At the sensitivity ≥ 0.95 operating point, 2 source-defined positive patients were missed, while 179 MSS patients were classified as positive. Decision curve analysis showed bootstrap-supported internal net-benefit superiority over the better default strategy only from approximately 0.055 through 0.30; the sensitivity ≥ 0.95 threshold of 0.0219 fell below this range. Moreover, these threshold and decision-curve analyses were derived from internally cross-validated mean-plus-max predictions and were not repeated in the reconstructed source-holdout setting. Together, these results support further investigation of the model as a candidate prioritization or pre-screening framework, but they do not justify withholding confirmatory testing or adopting the derived thresholds as clinical cutoffs.

The representation analyses indicated that aggregation design was an important component of model performance. Within the original fixed-fold cross-validation analysis, mean-plus-max pooling provided the most favorable overall numerical profile, whereas max-only pooling outperformed mean-only pooling. This ordering was not preserved under the reconstructed source holdout: mean-only pooling yielded a numerically higher ROC-AUC than mean-plus-max and max-only pooling, although paired bootstrap confidence intervals for the representation-level ROC-AUC differences included zero. The change in numerical ordering across validation schemes indicates that the apparent advantage of a particular pooling strategy was partition dependent and should not be interpreted as evidence of intrinsic representation superiority. The predominance of max-pooled dimensions among the highest-ranked SHAP features is therefore not sufficient to support a morphological multiple-instance-learning interpretation. Because raw max-pooled embeddings strongly encoded patch count, highly ranked max-pooled SHAP dimensions may also capture patch-burden or source-preprocessing proxies. Within the primary fixed-fold analysis, the combined representation performed more favorably than max pooling alone, consistent with additional information being retained by the mean-pooled component. However, the lack of the same numerical ordering in the source-holdout analysis indicates that the relative value of the pooling components was not invariant to dataset partitioning. Because the explanations remained confined to latent dimensions, they cannot establish that the model detected specific tumor regions, lymphocytic infiltration, mucinous morphology, or other recognizable MSI-associated histopathological patterns.

The patch-count analyses showed that interpretation of the max-pooled component requires additional caution. In the pooled cohort, source-defined positive patients had substantially more available patches than MSS patients, and patch count alone showed appreciable discrimination. The upstream source documentation indicates that the source TRAIN partition was patch-balanced by random reduction of the more numerous MSS training patches [16], providing a concrete provenance mechanism for at least part of the class-dependent patch-count structure. Because the probability of observing an extreme latent activation can increase with the number of available patches, part of the apparent advantage of max pooling may therefore reflect patch-burden or preprocessing structure rather than histopathological information alone. Consistent with this concern, performance was attenuated when a common fixed number of patches was imposed across eligible patients. Raw max-pooled embeddings also strongly encoded patch count in the out-of-fold decoding audit. After fold-contained residualization, useful multivariable patch-count decoding was substantially attenuated by R^2^ and mean absolute error, although a small inverse rank association remained. In the original random patient-level folds, the mean-plus-residualized-max representations still outperformed mean-only pooling; however, the source-holdout analysis did not confirm a transportable incremental advantage of max-containing representations over mean-only pooling. These findings indicate that patch burden contributed substantially to the raw max-pooled signal and that residualized random-fold performance should be interpreted cautiously rather than as proof that patch-count effects were fully removed.

The reconstructed source-partition analysis further showed that the pooled patch-count association was not stable across the original source structure. Patch-count discrimination was strong in source-TRAIN but approached chance level in source-TEST, consistent with the class-dependent patch-sampling history of the upstream training partition. Explicitly adding log1p-transformed patch count to the image-derived representations did not materially improve source-TEST discrimination. At the same time, image-derived representations retained moderate discrimination in source-TEST despite the loss of patch-count discrimination. These results argue against absolute patch burden alone explaining the source-holdout predictions, while also showing that the magnitude and pooling-specific pattern of performance under random patient-level cross-validation were sensitive to upstream source structure. Residual confounding by other source-associated technical, specimen-related, preprocessing-related, or morphological factors therefore remains possible.

In the separate proportional patch-subsampling analysis, performance also remained comparatively stable when patient representations were reconstructed from reduced patch fractions, without a monotonic benefit from increasing patch availability. This finding suggests that the model was not critically dependent on exhaustive patch enumeration within the existing archive and may have implications for computational efficiency. It does not, however, demonstrate robustness to differences in specimen sampling, staining, scanning, institutional practice, or external patch-generation pipelines.

Taken together, the findings support the methodological feasibility of a calibration-aware, embedding-based patient-level prediction framework while also demonstrating that validation design materially affects the estimated magnitude of performance. Random patient-level cross-validation yielded strong discrimination, whereas the reconstructed source holdout produced more moderate discrimination and revealed sensitivity to upstream dataset structure. Accordingly, the primary ROC-AUC of 0.915 should be interpreted as an estimate of performance under internal random patient-level partitioning rather than as evidence of source-independent generalizability. The source-holdout findings strengthen the rationale for independent multicenter validation rather than substituting for it. External multicenter validation, prospective workflow assessment, and spatially resolved pathological interpretation remain necessary before the framework can be considered for clinical integration.

### 4.2. Comparison with Previous MSI Prediction Studies

As outlined in the Introduction, Kather et al. first established the feasibility of inferring MSI-related information directly from routine H&E-stained gastrointestinal cancer histology [5]. Subsequent studies extended this concept through larger colorectal cancer cohorts, alternative deep-learning architectures, and multi-cohort validation designs [33]. Systematic reviews and meta-analytic evidence have since confirmed that histology-based MSI-H/dMMR prediction can achieve strong discrimination, while also documenting substantial heterogeneity related to cohort composition, reference standards, image processing, model design, and validation setting [4,34]. In particular, the lower and more variable specificity observed across external validation settings reinforces that favorable internal discrimination does not by itself establish clinical transportability.

Earlier MSI-prediction pipelines relied on task-specific convolutional training, patch-level classification, or trainable weakly supervised slide-level aggregation. The present study addressed a related but distinct question: whether a fixed pathology foundation model already encoded sufficient task-relevant information for patient-level prediction using deterministic pooling and comparatively simple downstream classifiers. Recent foundation-model benchmarks similarly suggest that performance depends not only on encoder capacity, but also on pretraining data, aggregation design, downstream adaptation, and validation strategy [23,24,35]. Consequently, neither increasing encoder scale nor using a more complex slide-level learner should be assumed to provide consistent improvements across cohorts and tasks.

The predictive performance observed in the present study lies within the broad range reported for histology-based MSI prediction, but direct numerical ranking against previous models would be inappropriate. Published studies differ in cancer-site composition, specimen type, MSI prevalence, molecular reference standard, tissue selection, preprocessing, and whether testing was performed internally or on genuinely independent cohorts [5,23,33,34]. More importantly, many previous investigations used PCR-, immunohistochemistry-, or sequencing-confirmed MSI-H/dMMR outcomes, whereas the current analysis used the source repository’s MSIMUT category, defined as microsatellite unstable or highly mutated, as the positive class. The available source metadata do not permit the highly mutated component to be assigned to a more specific molecular subgroup. The analytical target should therefore not be treated as equivalent to a strictly assay-confirmed MSI-H endpoint. Because the patch archive was derived from a TCGA-linked resource also represented in earlier MSI studies, the present analysis constitutes an internally validated methodological investigation rather than an independent external replication [5,16].

More clinically advanced studies have evaluated whether histology-based models can function as high-sensitivity pre-screening systems. MSIntuit was blindly validated in an independent consecutive cohort and designed to identify patients for whom standard MSI testing could potentially be avoided while maintaining very high sensitivity [36]. This operational logic is conceptually aligned with the sensitivity ≥ 0.95 operating point examined in Section 3.4 of the present study. However, the distinction in evidentiary strength is important: the MSIntuit thresholds were evaluated in an independent cohort and across different scanners, whereas the operating points reported here were derived and assessed internally and remain exploratory. A subsequent multicentre blinded evaluation involving more than 1200 patients further illustrates the level of external evidence required to assess cross-site transportability and realistic workflow performance [37].

The contribution of the present study therefore lies less in claiming higher discrimination than in characterizing dimensions of model behavior that have not been reported consistently across the MSI literature. The framework evaluated PR-AUC and probability accuracy alongside ROC-AUC, quantified calibration intercept, calibration slope, and calibration-in-the-large, compared calibration strategies within a leakage-safe nested procedure, examined prespecified sensitivity targets, and estimated decision-analytic net benefit. It also treated pooling strategy and patch availability as explicit methodological variables. These analyses provide a more complete account of internal model behavior than discrimination metrics alone, but they do not place the model at the same evidentiary level as externally validated or prospectively specified clinical systems.

The pooling and interpretability findings provide an additional point of comparison with trainable attention-based and multiple-instance-learning approaches. Rather than learning slide-level attention weights, the present framework compared deterministic mean, max, and mean-plus-max representations. The predominance of max-pooled dimensions among the highest-ranked SHAP features was compatible with predictive information being concentrated within a subset of patches, while the stronger overall performance of the combined representation within the random patient-level folds suggested that more distributed information remained complementary; however, this ordering was not preserved under the reconstructed source holdout. Because the analysis did not localize or pathologically annotate influential regions, it complements rather than replaces heatmap-based, attention-based, or pathologist-validated spatial interpretation used in previous studies.

Overall, the present study does not demonstrate superiority over previously published end-to-end, weakly supervised, foundation-model-based, or clinically validated MSI systems. Instead, it shows that fixed pathology foundation-model embeddings, deterministic patient-level pooling, and a parsimonious linear classifier can support informative internal prediction when accompanied by explicit evaluation of uncertainty, calibration, operating thresholds, decision-analytic value, and representation robustness. The study is therefore positioned as a methodological complement to prior MSI-prediction research and as a foundation for subsequent external multicentre and prospective validation.

### 4.3. Methodological and Translational Implications

The first methodological implication is that the analytical unit should correspond to the intended clinical unit of prediction. In patch-rich computational pathology datasets, the apparent number of image samples can obscure the fact that the effective number of independent observations is determined by patients. Maintaining patient-level separation throughout representation construction, model fitting, calibration, and evaluation was therefore essential to prevent correlated tissue fragments from producing optimistic estimates. Within this constraint, the combination of a fixed encoder, deterministic pooling, and a comparatively simple downstream classifier provided a modular framework in which representation, aggregation, and prediction could be examined separately.

This modular design has implications for reproducibility and model maintenance. Keeping the Phikon encoder fixed avoids repeated task-specific image-model training and allows downstream components to be audited, recalibrated, or replaced without retraining the complete image-processing pipeline. Related work in computational pathology has also illustrated the broader value of modular representation-transfer strategies, including the transfer of information from multimodal training frameworks to models operating on a single histopathology modality [21]. Recent pathology foundation-model benchmarks indicate that downstream performance depends not only on encoder capacity, but also on representation quality, aggregation strategy, task adaptation, and validation design [24,35]. In the present cohort, the favorable performance of logistic regression indicated that task-relevant information was accessible to a linear downstream classifier within the fixed Phikon-derived representation space. This finding should not be interpreted as evidence that simple classifiers will be optimal across other datasets, encoders, aggregation strategies, or clinical endpoints. Rather, it supports evaluating representation quality and aggregation design before increasing downstream model complexity.

An embedding-based workflow should nevertheless not be regarded as computationally cost-free. Whole-slide tiling, patch storage, and foundation-model inference may still impose substantial processing and infrastructure requirements. The principal efficiency advantage lies in avoiding repeated end-to-end task-specific training rather than eliminating whole-slide processing costs. Similarly, the stability observed under reduced patch fractions supports further investigation of predefined patch budgets, adaptive sampling, or sequential stopping strategies, but the present study did not measure execution time, storage requirements, energy consumption, or prospective turnaround time. The patch-burden findings therefore indicate the feasibility of resource-aware investigation rather than a demonstrated operational efficiency benefit.

A further translational implication is that discrimination must be complemented by evaluation of probability behavior and intended clinical action. Calibration analysis showed that informative rankings may coexist with probabilities requiring adjustment, while threshold analysis demonstrated that the relative consequences of missed positive cases and unnecessary confirmatory assessments vary substantially across operating points. Calibration and threshold selection should therefore be repeated in the intended deployment population, accounting for disease prevalence, clinical consequences, and workflow-specific tolerance for false-positive and false-negative results. The source-defined positive prevalence was 18.1% in the present cohort and may differ materially from that of future screening populations, with corresponding effects on predictive values and testing burden. Decision curve analysis can support this assessment but does not replace prospective workflow evaluation, health-economic analysis, or direct measurement of clinical outcomes [9,10,11,38].

The most plausible translational role for the framework is not autonomous MSI diagnosis, but candidate pre-screening and prioritization of confirmatory testing. An H&E-based probability estimate could potentially help identify cases for earlier molecular or immunohistochemical assessment, whereas a high-sensitivity operating point could be investigated within a rule-out-oriented workflow. If validated prospectively, such an approach might reduce avoidable testing, shorten diagnostic pathways, and, particularly in tissue-limited specimens, contribute to tissue stewardship by preserving tumor material for other clinically indicated molecular or targeted biomarker analyses. These potential benefits were not measured in the present study. Moreover, because the modeled endpoint was the source-defined MSIMUT category rather than a uniformly assay-confirmed MSI-H/dMMR outcome, the current framework cannot support withholding or replacing established PCR- or immunohistochemistry-based testing [36,37,38].

Representation-level interpretability provides only a partial basis for translational transparency. The SHAP analysis characterized reliance on latent mean- and max-pooled dimensions but did not identify the tissue structures responsible for individual predictions. For the max-pooled component, the patch producing the maximum value in a specific embedding dimension is mathematically traceable through dimension-wise argmax tracking, provided that patch-level embeddings and provenance mappings are retained. This could support future spatially resolved analyses, but it would identify dimension-specific source patches rather than, by itself, establish a single causal or pathologically meaningful region for the patient-level prediction. Future studies should combine such tracing with patient-level attribution, spatial visualization, pathologist review, and validation against recognizable histopathological patterns.

Clinical translation would additionally require external multicenter validation, locked preprocessing and calibration procedures, slide-quality control, assessment across scanners and staining protocols, evaluation of uncertain or non-evaluable cases, fairness analysis, and model-version governance. The present findings therefore support a transparent methodology for evaluating foundation-model embeddings in patient-level biomarker prediction, rather than a deployment-ready diagnostic system. Leakage control, modular representation design, probability calibration, operating-point analysis, decision-analytic assessment, and patch-burden testing address complementary requirements that should be considered before an embedding-based computational pathology model progresses toward prospective clinical evaluation.

### 4.4. Strengths and Limitations

This study has several methodological strengths. First, the patient was maintained as the analytical and validation unit throughout the complete workflow. Patch-level embeddings derived from the same patient were not divided between training and testing subsets, reducing the risk of optimistic performance estimates caused by correlated tissue fragments. A single verified patient manifest, outcome vector, representation matrix, and set of validation partitions were retained across classifier comparison, repeated validation, calibration, threshold analysis, pooling comparison, patch subsampling, and interpretability assessment. This consistency strengthened the internal traceability of the computational workflow.

Second, model evaluation extended beyond a single cross-validation estimate or discrimination metric. The primary five-fold analysis was complemented by repeated patient-level cross-validation, bootstrap uncertainty estimation, leakage-safe nested probability calibration, sensitivity-targeted operating-point analysis, and decision curve analysis with bootstrap-based uncertainty assessment. Additional sensitivity analyses examined clinicopathological and tissue-source-site structure within the cohort. Reporting ROC-AUC together with PR-AUC, Matthews correlation coefficient, Brier score, calibration parameters, predictive values, and confusion-matrix counts provided a broader characterization of model behavior under class imbalance. Pooling comparison and patch-burden experiments additionally evaluated assumptions concerning representation design and image availability, while SHAP analysis was explicitly restricted to representation-level interpretation. The use of a fixed encoder, deterministic pooling procedures, controlled random seeds, training-fold-contained preprocessing, and programmatically retained intermediate outputs further supported reproducibility and auditing.

Several limitations nevertheless constrain the interpretation of the findings. Most importantly, all analyses were based on a single retrospective TCGA-derived cohort, and no independent external dataset was available. Patient-level internal validation reduces information leakage but cannot reproduce the domain shifts encountered across independent institutions, patient populations, specimen-processing protocols, staining conditions, scanners, or digital-pathology systems. Two complementary source-structure assessments were included: TSS-group-isolated validation based on TCGA/GDC metadata and a reconstructed holdout based on the original upstream TRAIN/TEST partition. Both analyses remained internal to the same TCGA-derived source resource. The attenuation from a primary mean-plus-max ROC-AUC of 0.915 under random patient-level cross-validation to 0.788 under the reconstructed source holdout demonstrates that internal random-fold estimates were sensitive to source partitioning and should not be interpreted as evidence of transportability. Neither the TSS-isolated analysis nor the reconstructed source holdout constitutes independent external multicenter validation. The present results therefore characterize performance across multiple internal validation structures within the available source cohort rather than demonstrating transportability across clinical settings. Performance, calibration, fairness, and operating characteristics across independent patient populations, laboratories, staining workflows, scanners, and acquisition systems remain unknown.

The outcome definition also requires caution. The positive class remained the source repository’s MSIMUT category, formally defined as microsatellite unstable or highly mutated, rather than a uniformly clinically adjudicated PCR-, immunohistochemistry-, or sequencing-confirmed MSI-H/dMMR endpoint. GDC-derived MSIsensor2 status was available for all 360 cases. Among the 351 patients with an unambiguous GDC-derived MSI or MSS designation, agreement with the source archive label was 98.9%, with a Cohen’s κ of 0.959. Existing image-based out-of-sample predictions also retained strong discrimination when evaluated against this secondary GDC-derived endpoint. However, the GDC-derived MSIsensor2 annotation represents a bioinformatics-derived molecular annotation rather than a uniform clinical reference assay. The high concordance therefore supports consistency of the source labels but does not convert the primary outcome into a clinically adjudicated MSI-H/dMMR endpoint. Accordingly, the model should continue to be interpreted as predicting the source-defined archive label. Detailed MSIsensor2 concordance and secondary-endpoint performance results are provided in Supplementary Table S19.

The study also depended on a previously processed JPEG patch archive rather than direct reconstruction from the original whole-slide repository. Although the derivative dataset title refers to TCGA colon adenocarcinoma, linkage of all 360 patient identifiers to GDC metadata confirmed that the analytical cohort contained both TCGA-COAD and TCGA-READ cases, supporting its description as a colorectal cohort. Nevertheless, the derivative archive was accessed through a community-hosted repository without a persistent dataset DOI or a peer-reviewed data descriptor. The complete upstream case-selection pathway used to create the derivative archive—including the initial number of TCGA cases considered, case-level exclusion criteria, the number of originating whole-slide images, and all preprocessing decisions—could not be reconstructed fully. Upstream tissue detection, patch selection, stain normalization, slide-quality control, tiling stride, and overlap therefore could not be independently modified or completely audited. These provenance limitations remain relevant despite successful patient-level linkage to the original TCGA case identifiers.

The sample size was modest relative to the 1536-dimensional patient representation, and only 65 patients belonged to the source-defined positive class. Regularization, patient-level cross-validation, and repeated resampling reduced but did not eliminate risks related to high dimensionality and class imbalance. Regularization-sensitivity analysis further showed that discrimination remained similar across C = 0.01–1 but declined under substantially weaker regularization at C = 10–100, while no convergence warnings were observed across the evaluated configurations. Moreover, the candidate classifiers represented prespecified fixed implementations rather than an exhaustive or independently optimized comparison. Logistic regression and Linear SVM used class weighting, whereas the evaluated MLP, random forest, and gradient boosting implementations used unweighted objectives. Because imbalance handling was not harmonized across all candidate implementations, the classifier comparison should be interpreted as a comparison of the prespecified analytical pipelines rather than as an intrinsic ranking of the underlying algorithm families. The primary classifier was selected from five prespecified implementations using internal cross-validation within the same cohort. To examine potential classifier-selection optimism directly, a nested pipeline-selection sensitivity analysis restricted pipeline selection entirely to each outer-training set. Logistic regression was selected in all five outer folds, and the resulting nested-selected outer-test predictions were identical to those of the fixed logistic-regression reference, yielding no observed difference in ROC-AUC or PR-AUC. This finding supports the internal reproducibility of the original pipeline choice. Nevertheless, because both the original comparison and the nested sensitivity analysis were performed within the same cohort, model-selection uncertainty cannot be considered eliminated, and the results should not be interpreted as demonstrating intrinsic superiority of logistic regression over other algorithm families.

Calibration and decision-oriented analyses remained internal, although the principal source of calibration-method selection optimism identified in the original analysis was addressed through the fully nested calibration procedure. Calibration-method selection was repeated within a fully nested procedure in which uncalibrated probabilities, Platt scaling, and isotonic regression were compared using cross-fitted predictions generated exclusively from the corresponding outer-training data. Outer-test observations were not used for calibrator selection or fitting, and Platt scaling was selected in all five outer folds. The resulting nested-Platt probabilities showed substantially improved calibration, with a pooled calibration slope of 1.037 and calibration-in-the-large of 0.023. Nevertheless, calibration performance remains specific to the present development cohort and requires reassessment in an independent population. Similarly, the Youden and high-sensitivity operating thresholds were derived and evaluated from internally generated out-of-sample probabilities and remain exploratory cohort-specific operating points. Decision-curve analysis was supplemented by paired bootstrap uncertainty assessment, but its results likewise do not establish prospective clinical utility. The bootstrap procedures resampled patient-level outcome–prediction pairs without repeating the complete sequence of representation construction, model fitting, calibration selection, and threshold derivation and therefore quantify uncertainty conditional on the generated predictions rather than all sources of model-development uncertainty.

Interpretability remained confined to latent embedding dimensions. SHAP values characterized relative reliance on the mean- and max-pooled components but did not identify specific histopathological structures, cell populations, or causal biological mechanisms. The predominance of max-pooled dimensions among the top SHAP-ranked features should be interpreted cautiously because raw max-pooled embeddings strongly encoded patch count; these dimensions may therefore partly reflect patch-burden or source-preprocessing proxies. The interventional masker did not explicitly model correlations among embedding dimensions, and attribution magnitudes may differ under correlation-aware explanation methods. Dimension-wise max-pooling provenance could potentially permit tracing individual maximal activations back to source patches, but such tracing was not performed or validated through pathologist review. The original proportional patch-subsampling experiment represented repeated reductions within each patient’s existing archive and therefore assessed robustness to reduced patch availability, but it did not equalize absolute patch counts across patients and should not be interpreted as a control for patch-count confounding. Fixed-N, fold-contained patch-count-adjustment, source-holdout, and explicit patch-count-addition analyses demonstrated that absolute patch burden and upstream patch-generation history contributed to the raw max-pooled signal. Patch count itself may reflect a mixture of tissue area, tumor extent, source-image characteristics, and upstream patch-generation or tissue-selection processes; consequently, neither fixed-N subsampling nor statistical residualization can determine which biological or technical mechanism generated the observed patch-count difference.

Metadata linkage allowed several potentially relevant clinicopathological and site-related factors to be examined, including age, pathologic stage, tissue or organ of origin, primary diagnosis, TCGA project membership, and tissue-source-site code. These variables showed measurable associations with the source-defined outcome. A clinical-only model nevertheless discriminated less well than the image-only model, whereas combining the audited clinical variables with the image representation produced essentially no incremental discrimination over the image-only pipeline. In addition, discrimination was attenuated but remained substantial when all patients sharing the same TSS were isolated between training and testing. These findings indicate that the measured clinical and site variables do not fully account for the image-model discrimination; however, they do not exclude residual confounding by unmeasured morphological, technical, or clinical factors.

Important covariate limitations therefore remain. Sex/gender and a uniform clinical molecular MSI-test result were not included in the analyzable metadata extract used for the present sensitivity analyses, and the study did not incorporate pathologist-adjudicated histological subtype, detailed tumor-cell content, treatment information, complete pathology-report variables, or center-specific staining and scanner characteristics. Prospective turnaround time, computational cost, storage requirements, energy consumption, cost-effectiveness, tissue preservation, workflow acceptability, and effects on patient management were also not evaluated. A further reproducibility limitation is that the commit-specific revision of the Phikon encoder used during the original embedding-generation run was not pinned or retained; consequently, bitwise regeneration of the patch embeddings directly from the source images cannot be guaranteed across different hardware and software environments. This limitation is partly mitigated by the public release of the exact patient-specific embedding arrays used as fixed inputs for all downstream analyses. Collectively, these limitations indicate that the framework should be regarded as a transparent internally validated methodological investigation rather than a clinically validated or deployment-ready MSI decision-support system.

### 4.5. Future Directions

The immediate priority for future research is independent multicenter validation using a prespecified and frozen version of the complete analytical pipeline. The pathology foundation-model revision, preprocessing procedures, pooling strategy, classifier parameters, calibration mapping, and operating thresholds should be locked before external evaluation. Direct transportability should first be assessed without site-specific refitting or retrospective threshold optimization. If discrimination or calibration deteriorates, recalibration or domain-adaptation strategies should be evaluated in a separately designed development phase and tested in data that were not used to select the adaptation procedure. Performance should be reported by institution, scanner, staining protocol, specimen type, and clinically relevant patient subgroup to distinguish genuine generalizability from average performance that conceals site-specific failure [35,37,38,39].

Where transfer of raw whole-slide images is restricted by data-governance, infrastructure, or privacy constraints, distributed validation and federated-learning strategies could be investigated. The modular separation between fixed embedding extraction and a lightweight downstream classifier may facilitate such designs. However, latent embeddings should not be assumed to be inherently anonymous or privacy preserving. Any distributed implementation would require explicit evaluation of information leakage, secure aggregation, institutional governance, and reproducibility across local computational environments.

Future validation should also use clinically adjudicated reference standards. Cohorts with PCR-, immunohistochemistry-, or sequencing-confirmed MSI-H/dMMR status would determine whether the source-defined MSIMUT signal identified in the present study transfers to a more narrowly defined clinical endpoint. When multiple assays are available, discordant results should be reviewed explicitly rather than collapsed automatically into a binary label. Consecutive or population-based cohorts would additionally permit calibration, predictive values, testing burden, and operating thresholds to be evaluated under prevalence conditions that more closely reflect the intended clinical setting.

Robustness assessment should extend beyond random patch reduction within a single preprocessed archive. Relevant evaluations include alternative tissue blocks and slide sections from the same patient, biopsy versus resection material, variable tumor content, staining perturbations, scanner platforms, tissue-detection procedures, and external patch-generation pipelines. The modular framework also permits controlled comparison of Phikon with alternative pathology foundation-model encoders. Such comparisons should use identical patient partitions, pooling strategies, downstream classifiers, calibration procedures, and evaluation metrics to separate encoder-related differences from variation introduced elsewhere in the pipeline. Encoder selection should be completed within development data rather than through retrospective comparison on the final external test cohort.

Resource-aware processing should be evaluated concurrently with these robustness experiments. Predefined patch budgets, adaptive tissue sampling, uncertainty-guided patch selection, and sequential stopping criteria could be compared with exhaustive patch processing. These analyses should measure not only predictive performance but also inference time, storage requirements, computational cost, energy consumption, and diagnostic turnaround time. This would establish whether the observed internal tolerance to reduced patch fractions can produce a reproducible operational benefit.

Spatially resolved interpretability represents another important research direction. Dimension-wise provenance tracking could identify the source patches responsible for individual max-pooled activations, after which these patches could be mapped back to their originating slide locations. Patient-level attribution would then need to be integrated with spatial visualization and independent pathologist assessment. Such studies could examine whether influential regions correspond reproducibly to immune-rich areas, mucinous differentiation, glandular irregularity, architectural heterogeneity, or other recognizable histopathological patterns. Attribution stability across tissue perturbations, agreement among pathologists, and comparison with correlation-aware explanation methods would be required before latent feature importance could support morphological or biological interpretation.

Prospective studies should evaluate a clearly prespecified clinical role rather than generic deployment. A plausible initial setting would be model-assisted prioritization of confirmatory MSI testing, with molecular or immunohistochemical assessment remaining the reference procedure. A rule-out-oriented strategy would require externally validated sensitivity, a fixed operating threshold, and predefined management of uncertain, low-quality, and non-evaluable slides. Prospective outcomes should include missed positive cases, additional testing generated by false-positive predictions, turnaround time, tissue consumption, costs, workflow acceptability, and effects on clinical decisions. Retrospective threshold and decision-curve analyses can inform the design of such studies but cannot replace direct workflow evaluation [10,11,36,37,38].

Beyond their essential role in cohort description and subgroup assessment, clinicopathological variables should be further evaluated and expanded in independent external multimodal validation. In the internal sensitivity analysis, age, pathologic stage, tissue or organ of origin, primary diagnosis, TCGA project membership, and tissue-source-site information were examined, and combining the audited clinical variables with image-derived embeddings did not provide clear incremental discrimination over the image-only model. Future multimodal studies should therefore incorporate broader and prospectively collected variables that were unavailable or incompletely represented in the present cohort, including sex/gender, pathologist-adjudicated histological subtype, specimen type, structured pathology-report information, and center-specific technical factors. The incremental value of such multimodal integration should be tested against both image-only and clinical-only baselines in independent external cohorts using prespecified analytical pipelines, with particular attention to transportability, calibration, and clinical utility rather than model complexity alone.

Taken together, these directions define a staged translational pathway: first, they externally validate the locked image-based framework; second, assess clinically confirmed endpoints and domain robustness; third, connect latent representations with pathologist-recognizable morphology; and finally, evaluate a prespecified role within prospective clinical workflows. This sequence would determine whether foundation-model-derived histopathology embeddings can contribute safely, reproducibly, and efficiently to MSI pre-screening or confirmatory-test prioritization in colorectal cancer.

## 5. Conclusions

This study demonstrates the methodological feasibility of leakage-safe, patient-level prediction of a source-defined MSIMUT category in colorectal cancer histopathology using fixed Phikon foundation-model embeddings. Deterministic pooling of patch-level embeddings, followed by a comparatively simple logistic-regression classifier, supported strong internal discrimination under random patient-level cross-validation with the primary mean-plus-max representation (ROC-AUC, 0.915), but the same representation showed more moderate discrimination under the reconstructed source holdout (ROC-AUC, 0.788; PR-AUC, 0.593), indicating that the primary random-fold estimate was optimistic relative to source-holdout performance. Leakage-safe nested Platt calibration improved probability behavior without using outer-test outcomes for calibration-method selection, and the resulting calibrated predictions supported exploratory threshold and decision-curve analyses within the internally evaluated TCGA-derived cohort.

However, the additional provenance-based analyses materially qualify the interpretation of these findings. Reconstruction of the original source TRAIN/TEST partition showed that performance was attenuated under a source-structured internal holdout, with the mean-plus-max ROC-AUC decreasing to 0.788. Patch-count analyses further showed that absolute patch burden and upstream source-preprocessing history contributed to the raw max-pooled signal and to the magnitude of performance observed under random patient-level partitioning. Although image-derived representations retained moderate discrimination in source-TEST and explicit patch-count addition did not improve source-holdout performance, the results should not be interpreted as evidence of source-independent generalizability. The lower source-holdout performance was therefore considered a key limitation of the current framework and a major reason for restricting the interpretation to internal methodological feasibility.

The model should therefore be viewed as an internally evaluated methodological framework for MSI-related pre-screening research rather than as a clinically validated diagnostic tool. The predicted endpoint was the source-defined MSIMUT archive label, not a uniformly clinically adjudicated MSI-H/dMMR reference standard. All performance, calibration, threshold, decision-analytic, clinicopathological, source-provenance, and patch-count sensitivity results remain internal to the TCGA-derived cohort. Independent multicenter validation using clinically adjudicated MSI-H/dMMR outcomes, heterogeneous acquisition conditions, locked preprocessing and calibration procedures, and prespecified operating thresholds is required before any clinical application, confirmatory-testing decision, or workflow integration can be considered.

## Figures and Tables

**Table 1:** Overview of the verified study cohort and prespecified analytical design.

Category	Parameter	Value or Description
Study design	Design	Retrospective computational pathology study
Data source	Analyzed image archive	Kaggle-hosted “TCGA COAD MSI vs. MSS Prediction (JPG)” colorectal-only JPEG derivative
Data provenance	Upstream source	Kather et al. [16] FFPE histopathology dataset, Zenodo DOI: 10.5281/zenodo.2530835; derived from TCGA diagnostic slides
Data source	Data characteristics	Publicly available, de-identified, pre-extracted colorectal histopathology patch dataset
Eligibility	Inclusion requirement	Availability of image patches with an extractable TCGA patient identifier and a source-defined MSIMUT or MSS category label
Cohort	Number of patients	360
Cohort	MSIMUT-category patients	65 (18.1%)
Cohort	MSS-category patients	295 (81.9%)
Analytical structure	Primary analytical unit	Patient
Image input	Primary image modality	224 × 224-pixel JPEG patches derived from H&E-stained FFPE diagnostic whole-slide images
Image input	Source spatial resolution	0.5 µm/pixel
Image preprocessing	Source-level color normalization	Macenko normalization
Patch processing	Total analyzed patches	192,312
Feature extraction	Pretrained encoder	Original Phikon pathology foundation model (owkin/phikon)
Feature extraction	Patch embedding dimension	768
Patient representation	Pooling strategies	Mean pooling, max pooling, and mean-plus-max pooling
Patient representation	Mean-plus-max feature dimension	1536
Candidate models	Classifiers compared	Logistic Regression, Linear Support Vector Machine, Multilayer Perceptron, Random Forest, and Gradient Boosting
Validation	Primary validation	Five-fold patient-level cross-validation
Validation	Extended validation	Stratified five-fold cross-validation with 10 repeats
Uncertainty estimation	Bootstrap analysis	2000 resamples
Calibration	Approaches compared	Uncalibrated probabilities, Platt scaling, and isotonic regression
Operating-point assessment	Threshold analyses	Youden threshold and high-sensitivity operating points
Decision analysis	Method	Decision curve analysis
Robustness	Representation and image-burden analyses	Pooling comparison; proportional patch subsampling; fixed-N and patch-count-adjusted sensitivity analyses
Interpretability	Explanation level	Representation-level SHAP analysis

Abbreviations: FFPE, formalin-fixed paraffin-embedded; H&E, hematoxylin and eosin; MSI, microsatellite instability; MSIMUT, source-defined microsatellite-unstable or highly mutated category; MSS, microsatellite stable; SHAP, SHapley Additive exPlanations; TCGA, The Cancer Genome Atlas.

**Table 2:** Comparative performance of the candidate patient-level classifiers under fixed five-fold cross-validation.

Classifier	ROC-AUC	PR-AUC	Accuracy	Balanced Accuracy	Precision	Sensitivity	Specificity	F1	MCC	Brier
Logistic Regression	0.915 ± 0.035	0.809 ± 0.035	0.908	0.836	0.760	0.723	0.949	0.737	0.685	0.076
Linear SVM	0.911 ± 0.032	0.798 ± 0.042	0.894	0.768	0.799	0.569	0.966	0.654	0.613	0.077
Multi-Layer Perceptron	0.911 ± 0.032	0.803 ± 0.047	0.892	0.796	0.731	0.646	0.946	0.680	0.621	0.084
Random Forest	0.886 ± 0.025	0.696 ± 0.038	0.836	0.546	0.600	0.092	1.000	0.157	0.211	0.108
Gradient Boosting	0.826 ± 0.062	0.609 ± 0.113	0.847	0.631	0.792	0.292	0.969	0.386	0.389	0.129

Note: ROC-AUC and PR-AUC are reported as mean ± standard deviation across the five held-out folds. All remaining values are fold-wise means calculated at the default probability threshold of 0.50. No model-specific threshold optimization or post-hoc probability calibration was applied during candidate classifier comparison. MCC, Matthews correlation coefficient; PR-AUC, area under the precision–recall curve; ROC-AUC, area under the receiver operating characteristic curve; SVM, support vector machine.

**Table 3:** Repeated-validation and bootstrap-based internal performance estimates of the primary logistic regression model.

Metric	Repeated CV, Mean ± SD	Patient-Averaged Point Estimate	95% Bootstrap CI
ROC-AUC	0.911 ± 0.007	0.914	0.859–0.956
PR-AUC	0.797 ± 0.020	0.806	0.708–0.889
Accuracy	0.901 ± 0.008	0.900	0.867–0.931
Balanced accuracy	0.826 ± 0.013	0.831	0.772–0.886
Precision	0.739 ± 0.031	0.723	0.607–0.829
Sensitivity	0.709 ± 0.025	0.723	0.611–0.828
F1 score	0.718 ± 0.023	0.723	0.623–0.809
MCC	0.662 ± 0.028	0.662	0.552–0.764
Brier score	0.080 ± 0.006	0.071	0.051–0.095

Note: Repeated cross-validation results are reported as mean ± standard deviation across 10 repeat-level estimates. Performance was first averaged across the five held-out folds within each repeat. Patient-averaged point estimates were calculated after averaging the 10 out-of-sample probabilities available for each patient. The 95% confidence intervals were obtained from 2000 bootstrap resamples of the 360 patient-level outcome–probability pairs without refitting the prediction model. Threshold-dependent metrics were calculated at the default probability threshold of 0.50. CI, confidence interval; CV, cross-validation; MCC, Matthews correlation coefficient; PR-AUC, area under the precision–recall curve; ROC-AUC, area under the receiver operating characteristic curve; SD, standard deviation.

**Table 4:** Threshold-dependent performance of the nested-Platt-calibrated primary model.

Operating Strategy	Threshold	Accuracy	Balanced acc.	Sens.	Spec.	PPV	NPV	F1	MCC	TN	FP	FN	TP
Youden index	0.2474	0.878	0.865	0.846	0.885	0.618	0.963	0.714	0.652	261	34	10	55
Sensitivity ≥ 0.90	0.1327	0.808	0.847	0.908	0.786	0.484	0.975	0.631	0.564	232	63	6	59
Sensitivity ≥ 0.95	0.0219	0.497	0.681	0.969	0.393	0.260	0.983	0.410	0.297	116	179	2	63

Note: Thresholds were evaluated using the nested-selected Platt-calibrated out-of-sample probabilities. The Youden threshold maximized sensitivity plus specificity minus one. For each prespecified sensitivity target, the highest observed probability threshold satisfying the target was selected, thereby retaining the greatest achievable specificity among eligible thresholds. Thresholds are displayed to four decimal places, whereas all classifications and performance metrics were calculated using the corresponding full-precision probability values. These operating points were derived and evaluated using internally generated out-of-sample predictions from the same TCGA-derived cohort and therefore represent exploratory cohort-specific estimates rather than externally validated clinical cutoffs. FN, false negative; FP, false positive; MCC, Matthews correlation coefficient; NPV, negative predictive value; PPV, positive predictive value; TN, true negative; TP, true positive.

## Data Availability

The public Zenodo repository contains the non-identifying patient-specific Phikon patch-embedding arrays used in the study, pooled patient-level representation matrices, fixed cross-validation split definitions, documented random seeds and analysis configurations, out-of-sample model predictions, numerical analysis outputs, additional sensitivity-analysis outputs, reproducibility-audit files, and computational notebooks and analysis code. The Phikon encoder was loaded using the verified repository identifier owkin/phikon. However, a commit-specific model revision was not explicitly pinned or recorded during the original embedding-generation run, and the exact repository snapshot could not be verified retrospectively. Accordingly, no revision number is reported rather than assigning an unverified identifier. The exact embedding arrays generated during the original run are publicly archived and constitute the fixed inputs used for all downstream analyses, allowing those analyses to be reproduced without regenerating the embeddings. The original histopathology image files are not redistributed and remain available from their cited public sources. All derived materials and reproducibility resources are openly available at https://doi.org/10.5281/zenodo.22938170.

## Supplementary Materials

The following supporting information can be downloaded at https://www.mdpi.com/article/10.3390/diagnostics16203271/s1: Figure S1, Reliability assessment of pooled outer-test probabilities before and after leakage-safe nested Platt calibration; Table S1, Detailed cohort composition, patch-count distribution, and patient-level representation characteristics; Table S2, Detailed performance of candidate classifiers under fixed five-fold patient-level cross-validation; Table S3, Confusion-matrix-derived summaries for candidate classifiers; Table S4, Fold-wise confusion-matrix counts for the primary logistic regression model; Table S5, Fold-wise performance of the primary logistic regression model; Table S6, Repeated cross-validation and patient-level bootstrap uncertainty estimates; Table S7, Leakage-safe nested probability-calibration results and fold-wise method selection; Table S8, Threshold-based performance, bootstrap uncertainty, and testing burden of the nested-Platt primary model; Table S9, Complete numerical decision-curve analysis for the nested-Platt primary model; Table S10, Complete comparison of patient-level pooling strategies; Table S11, Patch-subsampling robustness under reduced patch availability; Table S12, Top 20 SHAP-ranked latent embedding dimensions; Table S13, Fold-wise SHAP contribution summary for the mean-pooled and max-pooled representation halves; Table S14, Fully nested candidate-pipeline selection sensitivity analysis; Table S15, Logistic-regression regularization and class-weight sensitivity analysis; Table S16, Clinicopathological and site-related associations with source-defined MSIMUT status; Table S17, Clinical-only, image-only, and image-plus-clinical model comparison; Table S18, Tissue-source-site-only and TSS-group-isolated sensitivity analyses; Table S19, GDC-derived MSIsensor2 concordance and secondary-endpoint image-model performance; Table S20, Fixed-N and patch-count-adjusted sensitivity analyses addressing absolute patch-burden confounding; Table S21, Source-partition holdout, explicit patch-count addition, and patch-count-decoding sensitivity analyses.

## Abbreviations

The following abbreviations are used in this manuscript:

**Abbreviation**	**Definition**
AJCC	American Joint Committee on Cancer
CI	Confidence interval
CITL	Calibration-in-the-large
COAD	Colon adenocarcinoma
CPU	Central processing unit
CUDA	Compute Unified Device Architecture
cuDNN	CUDA Deep Neural Network library
CV	Cross-validation
DCA	Decision curve analysis
dMMR	Deficient mismatch repair
FFPE	Formalin-fixed, paraffin-embedded
FN	False negative
FP	False positive
GDC	Genomic Data Commons
GPU	Graphics processing unit
H&E	Hematoxylin and eosin
MCC	Matthews correlation coefficient
MLP	Multilayer perceptron
MSI	Microsatellite instability
MSI-H	Microsatellite instability-high
MSIMUT	Source-defined microsatellite unstable or highly mutated category
MSS	Microsatellite stable
NPV	Negative predictive value
PCR	Polymerase chain reaction
PPV	Positive predictive value
PR-AUC	Area under the precision–recall curve
READ	Rectum adenocarcinoma
ROC-AUC	Area under the receiver operating characteristic curve
SD	Standard deviation
SHA-256	Secure Hash Algorithm 256-bit
SHAP	SHapley Additive exPlanations
SVM	Support vector machine
TCGA	The Cancer Genome Atlas
TN	True negative
TP	True positive
TRIPOD+AI	Transparent Reporting of a Multivariable Prediction Model for Individual Prognosis or Diagnosis–Artificial Intelligence
TSS	Tissue-source site
