# Supplementary material for: Calibration-Aware Patient-Level Prediction of Source-Defined MSIMUT Status in Colorectal Cancer from Whole-Slide Histopathology Images Using Foundation-Model Embeddings
Source: Diagnostics (Basel). 2026 Oct 9;16(20):3271. doi: 10.3390/diagnostics16203271 (PMC13703480; doi:10.3390/diagnostics16203271)
Supplement: Supplementary file 1 [file diagnostics-16-03271-s001.zip › diagnostics-4542710-supplementary.pdf]

Supplementary Materials

Calibration-Aware Patient-Level Prediction of Source-Defined MSIMUT Status in Colorectal Cancer from Whole-Slide Histopathology Images Using Foundation-Model Embeddings

Deniz Özkan Vardar [1] and Necati Vardar [2,*]

[1] Department of Pharmacy Services, Vocational School of Health Services, Lokman Hekim University, Ankara, Turkey; deniz.ozkanvardar@lokmanhekim.edu.tr

[2] Department of Electrical and Electronics Engineering, Faculty of Engineering and Natural Sciences, KTO Karatay University, Konya, Turkey; necati.vardar@karatay.edu.tr

[*] Correspondence: necati.vardar@karatay.edu.tr

This document contains one supplementary figure and twenty-one supplementary tables supporting the analyses reported in the main manuscript.

Supplementary Table S1. Detailed cohort composition, patch-count distribution, and patient-level representation characteristics.

Domain	Parameter	Value or Description
Cohort composition	Total number of patients	360
Cohort composition	Source-defined MSIMUT-category patients	65 (18.1%)
Cohort composition	MSS-category patients	295 (81.9%)
Patch archive	Total number of analyzed patches	192,312
Patch archive	Patches in the source-defined MSIMUT directory	75,039
Patch archive	Patches in the MSS directory	117,273
Patch-count distribution	Mean patches per patient	534.2
Patch-count distribution	Standard deviation	699.9
Patch-count distribution	Median patches per patient	291.5
Patch-count distribution	Interquartile range	119.8–592.3
Patch-count distribution	Minimum patches per patient	6
Patch-count distribution	Maximum patches per patient	4,305
Image input	Patch dimensions	224 × 224 pixels
Image input	Source spatial resolution	0.5 μm/pixel
Image input	Source-level color normalization	Macenko normalization
Feature extraction	Pretrained encoder	Original Phikon pathology foundation model (owkin/phikon)

Domain	Parameter	Value or Description
Feature extraction	Patch embedding dimension	768
Patient-level representation	Mean-pooled matrix dimensions	360 × 768
Patient-level representation	Max-pooled matrix dimensions	360 × 768
Patient-level representation	Mean-plus-max matrix dimensions	360 × 1,536
Patient-level representation	Primary analytical unit	Patient
Patient-level representation	Pooling order	Mean-pooled vector followed by max-pooled vector
Outcome definition	Positive analytical category	Source-defined MSIMUT category
Outcome definition	Negative analytical category	MSS
Outcome definition	Clinical interpretation	The source-defined MSIMUT category was not assumed to be equivalent to a uniformly assay-confirmed MSI-H/dMMR endpoint

Note: Patient groups were reconstructed from TCGA case identifiers embedded in the patch filenames. The source-defined MSIMUT category was used as the positive analytical outcome, whereas MSS was used as the negative outcome. The MSIMUT designation was inherited from the source repository and was not interpreted as a uniformly assay-confirmed MSI-H/dMMR endpoint. Patch counts describe the pre-extracted colorectal JPEG archive used in the present analysis. All model development, validation, calibration, threshold assessment, and interpretability analyses were conducted at the patient level. FFPE, formalin-fixed, paraffin-embedded; MSI-H, microsatellite instability-high; MSIMUT, source-defined microsatellite unstable or highly mutated category; MSS, microsatellite stable; TCGA, The Cancer Genome Atlas.

Candidate classifiers were evaluated using the same 1,536-dimensional mean-plus-max patient-level representation and the same fixed stratified five-fold patient-level cross-validation partitions. ROC-AUC and PR-AUC are reported as the mean and standard deviation across the five held-out folds. All remaining metrics are fold-wise means calculated at the default probability threshold of 0.50. No classifier-specific threshold optimization, post-hoc probability calibration, feature selection, dimensionality reduction, or data-driven hyperparameter search was performed during candidate comparison.

Supplementary Table S2. Detailed performance of candidate classifiers under fixed five-fold patient-level cross-validation.

Classifier	Mean ROC-AUC	SD ROC-AUC	Mean PR-AUC	SD PR-AUC	Accuracy	Balanced Accuracy	Precision	Sensitivity	F1 Score	MCC	Brier Score
Logistic Regression	0.914993	0.035424	0.809499	0.035248	0.908333	0.836115	0.759951	0.723077	0.736959	0.684743	0.076397
Linear SVM	0.911343	0.032398	0.797963	0.041878	0.894444	0.767666	0.799320	0.569231	0.654331	0.613056	0.077202
Multilayer Perceptron	0.911082	0.031634	0.803039	0.046674	0.891667	0.795958	0.730669	0.646154	0.680081	0.620882	0.084099
Random Forest	0.886050	0.024983	0.695710	0.038485	0.836111	0.546154	0.600000	0.092308	0.156905	0.211428	0.108127
Gradient Boosting	0.825815	0.061710	0.608744	0.112533	0.847222	0.630900	0.791667	0.292308	0.386022	0.389170	0.129357

Note: Logistic regression and the linear support vector machine used balanced class weights, whereas the evaluated multilayer perceptron, random forest, and gradient boosting implementations used unweighted objectives. The results therefore represent a comparison of the prespecified analytical pipelines and should not be interpreted as an intrinsic ranking of the underlying algorithm families. MCC, Matthews correlation coefficient; MLP, multilayer perceptron; PR-AUC, area under the precision–recall curve; ROC-AUC, area under the receiver operating characteristic curve; SD, standard deviation; SVM, support vector machine.

Average confusion-matrix-derived counts obtained for the five candidate classifiers under the fixed five-fold patient-level cross-validation framework. Values are reported as fold-wise means across the five held-out test folds and complement the classifier-level performance metrics reported in Supplementary Table S2.

Supplementary Table S3. Confusion-matrix-derived summaries for candidate classifiers.

Classifier	Mean TN	Mean FP	Mean FN	Mean TP
Logistic Regression	56.0	3.0	3.6	9.4
Linear SVM	57.0	2.0	5.6	7.4
Multilayer Perceptron	55.8	3.2	4.6	8.4
Random Forest	59.0	0.0	11.8	1.2
Gradient Boosting	57.2	1.8	9.2	3.8

Note: Each held-out fold contained 72 patients, including 13 patients from the source-defined positive category and 59 MSS patients. Values represent arithmetic means of the fold-specific confusion-matrix counts and therefore need not be integers. Threshold-dependent classification was performed at the default probability threshold of 0.50 without classifier-specific threshold optimization or post-hoc probability calibration. Metrics recalculated directly from the mean confusion counts may differ slightly from the fold-wise mean metrics reported in Supplementary Table S2 because nonlinear performance measures were calculated separately within each fold before averaging. FN, false negative; FP, false positive; MSS, microsatellite stable; TN, true negative; TP, true positive.

Fold-wise numbers of true-negative, false-positive, false-negative, and true-positive classifications obtained for the selected logistic regression model under the fixed five-fold patient-level cross-validation framework. Each patient appeared in exactly one held-out test fold, and no patient contributed observations to both the training and test portions of the same fold.

Supplementary Table S4. Fold-wise confusion-matrix counts for the primary logistic regression model.

Fold	Training Patients, n	Test Patients, n	TN	FP	FN	TP
1	288	72	57	2	2	11
2	288	72	56	3	3	10
3	288	72	55	4	4	9
4	288	72	55	4	3	10
5	288	72	57	2	6	7
**Mean**	**288**	**72**	**56.0**	**3.0**	**3.6**	**9.4**
**Total held-out classifications**	—	**360**	**280**	**15**	**18**	**47**

Note: Each held-out test fold contained 72 patients, including 59 MSS patients and 13 patients from the source-defined positive category. Classification was performed at the default probability threshold of 0.50 without post-hoc probability calibration or classifier-specific threshold optimization. The mean row reports arithmetic means across the five held-out folds. The total held-out classifications row aggregates the mutually exclusive test-fold predictions across all 360 patients and should not be confused with the patient-averaged predictions obtained from repeated cross-validation. FN, false negative; FP, false positive; MSS, microsatellite stable; TN, true negative; TP, true positive.

Fold-wise discrimination, classification, and probability-performance metrics obtained for the selected logistic regression model under the fixed five-fold patient-level cross-validation framework. ROC-AUC and PR-AUC quantify threshold-independent discrimination. Accuracy, balanced accuracy, precision, sensitivity, F1 score, and Matthews correlation coefficient were calculated at the default probability threshold of 0.50. The Brier score summarizes the mean squared error of the predicted probabilities.

Supplementary Table S5. Fold-wise performance of the primary logistic regression model.

Fold	Training Patients, n	Test Patients, n	ROC-AUC	PR-AUC	Accuracy	Balanced Accuracy	Precision	Sensitivity	F1 Score	MCC	Brier Score
1	288	72	0.929596	0.815389	0.944444	0.906128	0.846154	0.846154	0.846154	0.812256	0.058467
2	288	72	0.899609	0.838185	0.916667	0.859192	0.769231	0.769231	0.769231	0.718383	0.075075
3	288	72	0.863103	0.808419	0.888889	0.812256	0.692308	0.692308	0.692308	0.624511	0.085212
4	288	72	0.956975	0.834882	0.902778	0.850717	0.714286	0.769231	0.740741	0.681721	0.068456
5	288	72	0.925684	0.750620	0.888889	0.752282	0.777778	0.538462	0.636364	0.586843	0.094776
**Mean**	**288**	**72**	**0.914993**	**0.809499**	**0.908333**	**0.836115**	**0.759951**	**0.723077**	**0.736959**	**0.684743**	**0.076397**
**SD**	—	—	**0.035424**	**0.035248**	—	—	—	—	—	—	—

Note: All preprocessing steps, including median imputation and feature standardization, were fitted exclusively within the corresponding training fold. Logistic regression was fitted using balanced class weights, a maximum of 5,000 iterations, and a fixed random seed of 42. No post-hoc probability calibration, classifier-specific threshold optimization, feature selection, dimensionality reduction, or data-driven hyperparameter search was performed during this fixed-fold candidate-comparison stage. ROC-AUC and PR-AUC are summarized as the mean and standard deviation across the five held-out folds; the remaining summary values represent arithmetic means of the fold-specific metrics. MCC, Matthews correlation coefficient; PR-AUC, area under the precision–recall curve; ROC-AUC, area under the receiver operating characteristic curve; SD, standard deviation.

Repeated-validation and bootstrap-based internal performance estimates for the primary logistic regression model. Repeated cross-validation results summarize 10 repeat-level estimates obtained from stratified five-fold patient-level cross-validation. Within each repeat, performance was first averaged across the five held-out folds. Patient-averaged point estimates were calculated after averaging the 10 out-of-sample probability estimates available for each patient. Two-sided 95% confidence intervals were obtained from 2,000 nonparametric bootstrap resamples of the 360 patient-level outcome–probability pairs.

Supplementary Table S6. Repeated cross-validation and patient-level bootstrap uncertainty estimates.

Metric	Repeated CV, mean ± SD	Patient-averaged point estimate	95% bootstrap CI
ROC-AUC	0.911082 ± 0.007176	0.913638	0.859445–0.955836
PR-AUC	0.797034 ± 0.019579	0.805630	0.707708–0.889183
Accuracy	0.900556 ± 0.008466	0.900000	0.866667–0.930556
Balanced accuracy	0.825971 ± 0.013285	0.831030	0.771698–0.886295
Precision	0.738639 ± 0.031267	0.723077	0.607128–0.828978
Sensitivity	0.709231 ± 0.024540	0.723077	0.611111–0.828125
F1 score	0.718054 ± 0.023278	0.723077	0.622951–0.808824
MCC	0.662120 ± 0.028302	0.662060	0.551532–0.764001
Brier score	0.080177 ± 0.005895	0.071457	0.051150–0.095190

Note: Repeated cross-validation results are reported as the mean and standard deviation across 10 repeat-level estimates rather than across the 50 individual fold evaluations. This two-stage summarization avoided treating folds within the same repeat as statistically independent observations. Patient-averaged point estimates were calculated from one averaged out-of-sample probability per patient. Bootstrap resampling was performed at the patient level using 2,000 valid resamples and a fixed random seed of 42. The prediction model was not refitted during bootstrap analysis; the confidence intervals therefore quantify uncertainty conditional on the generated patient-averaged out-of-sample predictions and do not incorporate all sources of model-development uncertainty. Threshold-dependent metrics were calculated at the default probability threshold of 0.50. CI, confidence interval; CV, cross-validation; MCC, Matthews correlation coefficient; PR-AUC, area under the precision–recall curve; ROC-AUC, area under the receiver operating characteristic curve; SD, standard deviation.

Supplementary Table S7. Leakage-safe nested probability-calibration results and fold-wise method selection.

Calibration was re-evaluated using a fully nested patient-level procedure. Within each outer fold, calibration-method selection was based only on cross-fitted predictions generated from the corresponding outer-training data; the selected calibration mapping was then fitted without access to the outer-test outcomes and applied to the untouched outer-test fold. Pooled metrics below were calculated after concatenating the mutually exclusive outer-test predictions from all five folds.

Panel A. Pooled outer-test probability and calibration metrics

Method	ROC-AUC	PR-AUC	Brier	Cal. intercept	Cal. slope	CITL	Nested selected?
Uncalibrated	0.915	0.802	0.076	-0.407	0.403	0.040	No
Platt scaling	0.913	0.796	0.073	0.057	1.037	0.023	Yes (5/5)
Isotonic regression	0.901	0.776	0.076	-0.341	0.687	0.025	No

Note: The joint calibration intercept and slope were estimated from the pooled outer-test probabilities. Calibration-in-the-large (CITL) was reported separately. Platt scaling improved the pooled Brier score from 0.076 to 0.073 and yielded a calibration slope close to 1. Isotonic regression produced tied probability values and was not selected for downstream analyses. CITL, calibration-in-the-large; PR-AUC, area under the precision–recall curve; ROC-AUC, area under the receiver operating characteristic curve.

Within each outer-training set, uncalibrated, Platt-scaled, and isotonic probabilities were compared using inner cross-validated Brier score. The lowest-Brier method was selected separately in every outer fold.

Panel B. Inner cross-validation calibration-method selection by outer fold

Outer fold	Inner Brier: uncalibrated	Inner Brier: Platt	Inner Brier: isotonic	Selected method
1	0.0842	0.0795	0.0910	Platt scaling
2	0.0864	0.0838	0.0880	Platt scaling
3	0.0790	0.0739	0.0774	Platt scaling
4	0.0901	0.0823	0.0900	Platt scaling
5	0.0799	0.0769	0.0793	Platt scaling

Note: Platt scaling was selected in all five outer folds using outer-training data only. A same-score audit confirmed that Platt scaling preserved ROC-AUC exactly within each individual outer fold because it is a strictly monotonic transformation of the base logistic-regression score. The small difference between pooled uncalibrated and pooled Platt ROC-AUC values reflects concatenation of fold-specific probability mappings rather than within-fold rank changes. The nested-selected Platt probabilities were carried forward to Supplementary Tables S8 and S9 and Figure S1.

Threshold-dependent classification performance was recalculated using the pooled leakage-safe nested-Platt outer-test probabilities. The Youden operating point maximized sensitivity plus specificity minus one. For each prespecified sensitivity target, the highest observed probability threshold meeting the target was selected. Patient-level stratified bootstrap resampling was used to quantify fixed-threshold uncertainty.

Supplementary Table S8. Threshold-based performance, bootstrap uncertainty, and testing burden of the nested-Platt primary model.

Operating strategy	Threshold	Accuracy	Balanced Accuracy	Sensitivity	Specificity	PPV	NPV	F1 Score	MCC	TN	FP	FN	TP
Youden index	0.2474	0.878	0.865	0.846	0.885	0.618	0.963	0.714	0.652	261	34	10	55
Sensitivity ≥0.90	0.1327	0.808	0.847	0.908	0.786	0.484	0.975	0.631	0.564	232	63	6	59
Sensitivity ≥0.95	0.0219	0.497	0.681	0.969	0.393	0.260	0.983	0.410	0.297	116	179	2	63

Panel B. Patient-level stratified bootstrap 95% confidence intervals for fixed operating thresholds

Operating strategy	Sensitivity	Specificity	PPV	NPV	Accuracy	Balanced accuracy	F1	MCC
Youden index	0.846 (0.754–0.923)	0.885 (0.847–0.919)	0.618 (0.545–0.700)	0.963 (0.941–0.981)	0.878 (0.844–0.911)	0.865 (0.814–0.910)	0.714 (0.644–0.783)	0.652 (0.561–0.736)
Sensitivity ≥0.90	0.908 (0.831–0.969)	0.786 (0.739–0.834)	0.484 (0.429–0.545)	0.975 (0.954–0.992)	0.808 (0.767–0.847)	0.847 (0.801–0.886)	0.631 (0.573–0.689)	0.564 (0.485–0.636)
Sensitivity ≥0.95	0.969 (0.923–1.000)	0.393 (0.339–0.447)	0.260 (0.242–0.281)	0.983 (0.957–1.000)	0.497 (0.450–0.542)	0.681 (0.645–0.714)	0.410 (0.385–0.437)	0.297 (0.243–0.344)

Panel C. Confirmatory-testing burden per 100 patients with bootstrap 95% confidence intervals

Operating strategy	Confirmatory tests /100	Tests avoided /100 vs test-all	Source-defined positives missed /100
Youden index	24.72 (21.39–28.06)	75.28 (71.94–78.61)	2.78 (1.39–4.44)
Sensitivity ≥0.90	33.89 (29.72–37.78)	66.11 (62.22–70.28)	1.67 (0.56–3.06)
Sensitivity ≥0.95	67.22 (62.50–71.67)	32.78 (28.33–37.50)	0.56 (0.00–1.39)

Note: Patients with predicted probabilities greater than or equal to the selected threshold were classified as positive for the source-defined MSIMUT category. Thresholds are displayed to four decimal places, whereas all classifications were calculated from full-precision values. Bootstrap intervals in Panels B and C were obtained by patient-level stratified resampling while holding the observed operating threshold fixed. These internally derived thresholds quantify cohort-specific screening trade-offs and are not externally validated clinical cutoffs. MCC, Matthews correlation coefficient; NPV, negative predictive value; PPV, positive predictive value; TN, true negative; TP, true positive.

Complete threshold-specific decision-curve results for the nested-Platt primary logistic regression model and the test-all and test-none reference strategies. Decision curve analysis was evaluated from threshold probability 0.01 to 0.30 in increments of 0.005. Patient-level stratified bootstrap resampling quantified uncertainty in model net benefit and in the paired difference between the model and the better default strategy at each threshold.

Supplementary Table S9. Complete numerical decision-curve analysis for the nested-Platt primary model.

Threshold	Model net benefit	Test-all net benefit	Test-none net benefit	Δ vs best default	95% CI for Δ	TP	FP	FN	TN	Model > both defaults	Lower 95% > best default
0.010	0.171605	0.172278	0.000000	-0.000673	-0.006453–0.002413	64	220	1	75	No	No
0.015	0.169360	0.168077	0.000000	0.001283	-0.004654–0.004569	64	199	1	96	Yes	No
0.020	0.167460	0.163832	0.000000	0.003628	-0.002382–0.007086	64	182	1	113	Yes	No
0.025	0.157336	0.159544	0.000000	-0.002208	-0.013535–0.006909	61	170	4	125	No	No
0.030	0.155613	0.155212	0.000000	0.000401	-0.011142–0.009593	61	161	4	134	Yes	No
0.035	0.154231	0.150835	0.000000	0.003397	-0.008022–0.012637	61	151	4	144	Yes	No
0.040	0.153588	0.146412	0.000000	0.007176	-0.004167–0.016667	61	137	4	158	Yes	No
0.045	0.149651	0.141943	0.000000	0.007708	-0.005267–0.018691	60	130	5	165	Yes	No
0.050	0.149561	0.137427	0.000000	0.012135	-0.001023–0.023249	60	117	5	178	Yes	No
0.055	0.148236	0.132863	0.000000	0.015373	0.001998–0.026808	60	114	5	181	Yes	Yes
0.060	0.146809	0.128251	0.000000	0.018558	0.005198–0.030024	60	112	5	183	Yes	Yes
0.065	0.146197	0.123589	0.000000	0.022608	0.009180–0.034033	60	106	5	189	Yes	Yes
0.070	0.146177	0.118877	0.000000	0.027300	0.013825–0.038771	60	98	5	197	Yes	Yes
0.075	0.145946	0.114114	0.000000	0.031832	0.018243–0.043393	60	92	5	203	Yes	Yes
0.080	0.143116	0.109300	0.000000	0.033816	0.020290–0.046380	59	86	6	209	Yes	Yes
0.085	0.142213	0.104432	0.000000	0.037781	0.024150–0.050501	59	84	6	211	Yes	Yes

Threshold	Model net benefit	Test-all net benefit	Test-none net benefit	Δ vs best default	95% CI for Δ	TP	FP	FN	TN	Model > both defaults	Lower 95% > best default
0.090	0.142186	0.099512	0.000000	0.042674	0.028815–0.055464	59	79	6	216	Yes	Yes
0.095	0.141436	0.094537	0.000000	0.046900	0.033011–0.059914	59	77	6	218	Yes	Yes
0.100	0.140741	0.089506	0.000000	0.051235	0.037346–0.064198	59	75	6	220	Yes	Yes
0.105	0.139773	0.084420	0.000000	0.055354	0.041620–0.068269	59	74	6	221	Yes	Yes
0.110	0.138826	0.079276	0.000000	0.059551	0.045974–0.072691	59	73	6	222	Yes	Yes
0.115	0.138622	0.074074	0.000000	0.064548	0.050547–0.077715	59	70	6	225	Yes	Yes
0.120	0.138510	0.068813	0.000000	0.069697	0.055552–0.082828	59	67	6	228	Yes	Yes
0.125	0.138889	0.063492	0.000000	0.075397	0.061111–0.088492	59	63	6	232	Yes	Yes
0.130	0.137739	0.058110	0.000000	0.079630	0.065485–0.092819	59	63	6	232	Yes	Yes
0.135	0.133799	0.052665	0.000000	0.081134	0.065768–0.095279	58	63	7	232	Yes	Yes
0.140	0.134432	0.047158	0.000000	0.087274	0.071641–0.101552	58	59	7	236	Yes	Yes
0.145	0.134730	0.041585	0.000000	0.093145	0.077794–0.107554	58	56	7	239	Yes	Yes
0.150	0.134150	0.035948	0.000000	0.098203	0.082349–0.112749	58	55	7	240	Yes	Yes
0.155	0.130819	0.030243	0.000000	0.100575	0.084466–0.115763	57	54	8	241	Yes	Yes
0.160	0.130291	0.024471	0.000000	0.105820	0.089679–0.120899	57	53	8	242	Yes	Yes
0.165	0.130339	0.018629	0.000000	0.111710	0.095558–0.127212	57	51	8	244	Yes	Yes
0.170	0.129317	0.012718	0.000000	0.116600	0.100368–0.132129	57	51	8	244	Yes	Yes
0.175	0.128872	0.006734	0.000000	0.122138	0.105892–0.137374	57	50	8	245	Yes	Yes
0.180	0.127846	0.000678	0.000000	0.127168	0.110840–0.142547	57	50	8	245	Yes	Yes
0.185	0.126551	-0.005453	0.000000	0.126551	0.109765–0.142706	56	46	9	249	Yes	Yes

Threshold	Model net benefit	Test-all net benefit	Test-none net benefit	Δ vs best default	95% CI for Δ	TP	FP	FN	TN	Model > both defaults	Lower 95% > best default
0.190	0.126886	-0.011660	0.000000	0.126886	0.109911–0.143210	56	44	9	251	Yes	Yes
0.195	0.125949	-0.017943	0.000000	0.125949	0.108782–0.142443	56	44	9	251	Yes	Yes
0.200	0.125000	-0.024306	0.000000	0.125000	0.107639–0.141667	56	44	9	251	Yes	Yes
0.205	0.126188	-0.030748	0.000000	0.126188	0.108630–0.142769	56	41	9	254	Yes	Yes
0.210	0.125281	-0.037271	0.000000	0.125281	0.107525–0.141777	56	41	9	254	Yes	Yes
0.215	0.124363	-0.043878	0.000000	0.124363	0.106664–0.141031	56	41	9	254	Yes	Yes
0.220	0.120655	-0.050570	0.000000	0.120655	0.102493–0.137687	55	41	10	254	Yes	Yes
0.225	0.120520	-0.057348	0.000000	0.120520	0.102151–0.138085	55	40	10	255	Yes	Yes
0.230	0.122078	-0.064214	0.000000	0.122078	0.103499–0.139827	55	37	10	258	Yes	Yes
0.235	0.122912	-0.071169	0.000000	0.122912	0.105169–0.140214	55	35	10	260	Yes	Yes
0.240	0.122076	-0.078216	0.000000	0.122076	0.104094–0.139474	55	35	10	260	Yes	Yes
0.245	0.121229	-0.085357	0.000000	0.121229	0.102980–0.138723	55	35	10	260	Yes	Yes
0.250	0.118519	-0.092593	0.000000	0.118519	0.099074–0.137037	54	34	11	261	Yes	Yes
0.255	0.114896	-0.099925	0.000000	0.114896	0.094349–0.134415	53	34	12	261	Yes	Yes
0.260	0.114039	-0.107357	0.000000	0.114039	0.093315–0.133634	53	34	12	261	Yes	Yes
0.265	0.113171	-0.114890	0.000000	0.113171	0.092271–0.132842	53	34	12	261	Yes	Yes
0.270	0.110540	-0.122527	0.000000	0.110540	0.088432–0.130596	52	33	13	262	Yes	Yes
0.275	0.113889	-0.130268	0.000000	0.113889	0.092615–0.133716	52	29	13	266	Yes	Yes
0.280	0.114198	-0.138117	0.000000	0.114198	0.092735–0.133650	52	28	13	267	Yes	Yes
0.285	0.114549	-0.146076	0.000000	0.114549	0.093454–0.134538	52	27	13	268	Yes	Yes

Threshold	Model net benefit	Test-all net benefit	Test-none net benefit	Δ vs best default	95% CI for Δ	TP	FP	FN	TN	Model > both defaults	Lower 95% > best default
0.290	0.109390	-0.154147	0.000000	0.109390	0.087440–0.130213	50	26	15	269	Yes	Yes
0.295	0.110993	-0.162333	0.000000	0.110993	0.089416–0.131600	50	24	15	271	Yes	Yes
0.300	0.107540	-0.170635	0.000000	0.107540	0.086111–0.128968	49	24	16	271	Yes	Yes

Note: Net benefit was calculated as TP/n − (FP/n) × pt/(1 − pt), where n is the number of patients and pt is the threshold probability. Test-all net benefit used the observed source-defined positive prevalence of 65/360; test-none net benefit was zero. The "best default" was the larger of test-all and test-none at each threshold. The model's observed net benefit exceeded both defaults over 0.015–0.020 and 0.030–0.300, with brief exceptions at 0.010 and 0.025; the lower 2.5th percentile of the paired bootstrap difference exceeded the better default from 0.055 through 0.300. These results are exploratory internal decision-analytic estimates and do not establish prospective clinical utility, cost-effectiveness, or safety of withholding confirmatory testing.

Performance comparison of the three prespecified deterministic patient-level pooling strategies. Mean pooling summarized the average activation of each latent embedding dimension across all available patches for a patient, max pooling retained the largest activation observed for each dimension and mean-plus-max pooling concatenated the corresponding mean-pooled and max-pooled vectors. All representations were evaluated using the same primary logistic regression classifier and the same fixed five-fold patient-level cross-validation partitions.

Supplementary Table S10. Complete comparison of patient-level pooling strategies.

Pooling Strategy	Representation Dimension	Mean ROC-AUC	SD ROC-AUC	Mean PR-AUC	SD PR-AUC	Accuracy	Balanced Accuracy	Precision	Sensitivity	Specificity	NPV	F1 Score	MCC	Brier Score
Mean-plus-max pooling	1,536	0.914993	0.035424	0.809499	0.035248	0.908333	0.836115	0.759951	0.723077	0.949153	0.940099	0.736959	0.684743	0.076397
Max pooling	768	0.911864	0.035629	0.767117	0.062199	0.888889	0.800261	0.712857	0.661538	0.938983	0.926803	0.681589	0.618457	0.085225
Mean pooling	768	0.820078	0.046490	0.591972	0.075696	0.830556	0.728683	0.523343	0.569231	0.888136	0.905132	0.535078	0.440330	0.140686

Note: Mean and max pooling each produced a 768-dimensional patient-level representation, whereas concatenated mean-plus-max pooling produced a 1,536-dimensional representation. Logistic regression was evaluated using balanced class weights, a maximum of 5,000 iterations, and a fixed random seed of 42. All preprocessing operations were fitted exclusively within the corresponding training fold. ROC-AUC and PR-AUC are reported as fold-wise mean and standard deviation. Threshold-dependent metrics were calculated at the default probability threshold of 0.50 and are reported as arithmetic means across the five held-out folds. No post-hoc probability calibration, threshold optimization, feature selection, dimensionality reduction, or data-driven hyperparameter search was performed in the pooling comparison. MCC, Matthews correlation coefficient; NPV, negative predictive value; PR-AUC, area under the precision–recall curve; ROC-AUC, area under the receiver operating characteristic curve; SD, standard deviation.

Performance of the primary mean-plus-max logistic regression pipeline under reduced patch availability. For each evaluated fraction, patch embeddings were randomly subsampled without replacement within each patient, patient-level mean-plus-max representations were reconstructed, and the primary classifier was evaluated using the same fixed patient-level cross-validation framework.

Supplementary Table S11. Patch-subsampling robustness under reduced patch availability.

Patch fraction retained per patient	Mean ROC-AUC	SD ROC-AUC	Mean PR-AUC	SD PR-AUC
25%	0.914654	0.005450	0.793534	0.014513
50%	0.918931	0.004480	0.806677	0.007938
75%	0.915072	0.002790	0.803725	0.007909
100%	0.914993	0.000000	0.809499	0.000000

Note: Patch fractions indicate the proportion of each patient's available patch embeddings retained before reconstruction of the mean-plus-max patient-level representation. Subsampling was performed independently within each patient without replacement and was repeated 10 times per fraction. For each patch fraction and repetition, performance was first averaged across the five held-out folds. The reported means and standard deviations were then calculated across the 10 repeat-level estimates. At the 100% condition, all available patches were retained, and the reconstructed patient-level representations were identical across repetitions; consequently, repeat-level standard deviations were zero by construction. Differences among fractions were interpreted descriptively, and no formal pairwise superiority testing was performed. Patch subsampling simulated reduced patch availability within the existing archive and should not be interpreted as validation on limited-biopsy specimens, externally acquired slides, alternative tissue-processing protocols, or different scanning systems. PR-AUC, area under the precision–recall curve; ROC-AUC, area under the receiver operating characteristic curve; SD, standard deviation.

The 20 highest-ranking latent embedding dimensions according to mean absolute SHAP contribution in the fold-wise out-of-sample interpretability analysis of the primary logistic regression model. Feature labels identify both the pooling operation and the corresponding latent Phikon embedding dimension.

Supplementary Table S12. Top 20 SHAP-ranked latent embedding dimensions.

Rank	Feature Label	Global Feature Index	Latent Dimension	Representation Half	Mean Absolute SHAP Value
1	max_246	1013	246	Max-pooled	0.150480
2	max_522	1289	522	Max-pooled	0.136758
3	mean_200	199	200	Mean-pooled	0.128095
4	max_200	967	200	Max-pooled	0.127794
5	max_37	804	37	Max-pooled	0.126995
6	max_573	1340	573	Max-pooled	0.120415
7	max_451	1218	451	Max-pooled	0.118795
8	mean_396	395	396	Mean-pooled	0.118603
9	max_390	1157	390	Max-pooled	0.113191
10	max_27	794	27	Max-pooled	0.112988
11	mean_621	620	621	Mean-pooled	0.109618
12	max_758	1525	758	Max-pooled	0.109034
13	max_91	858	91	Max-pooled	0.108685
14	max_206	973	206	Max-pooled	0.105673
15	mean_48	47	48	Mean-pooled	0.100161
16	max_201	968	201	Max-pooled	0.098084
17	max_306	1073	306	Max-pooled	0.097118
18	max_244	1011	244	Max-pooled	0.097085
19	max_109	876	109	Max-pooled	0.096970
20	max_295	1062	295	Max-pooled	0.096873

Note: SHAP values were calculated for the final 1,536-dimensional mean-plus-max patient-level representation. The first 768 global feature positions corresponded to the mean-pooled representation, and the second 768 positions corresponded to the max-pooled representation. Feature labels use one-based latent-dimension numbering, whereas the global feature index reflects the zero-based column position used in the analytical feature matrix. For example, mean_200 corresponds to global feature index 199, whereas max_200 corresponds to global feature index 967. Mean absolute SHAP values quantify the average magnitude of each feature's contribution to the model output across held-out patients and do not indicate the direction of contribution. The latent dimensions are not directly mapped to specific histological structures, cell types, or tissue regions. SHAP, SHapley Additive exPlanations.

Fold-wise comparison of mean absolute SHAP contributions aggregated across the mean-pooled and max-pooled halves of the patient-level representation. SHAP contributions were calculated from held-out patient predictions within each fold of the fixed five-fold patient-level cross-validation framework.

Supplementary Table S13. Fold-wise SHAP contribution summary for the mean-pooled and max-pooled representation halves.

Fold	Training Patients, n	Test Patients, n	Mean Absolute SHAP Contribution, Mean-Pooled Half	Mean Absolute SHAP Contribution, Max-Pooled Half	Absolute Difference, Max − Mean	Max/Mean Contribution Ratio
1	288	72	0.029589	0.038482	0.008893	1.301
2	288	72	0.028777	0.037660	0.008883	1.309
3	288	72	0.028969	0.036768	0.007799	1.269
4	288	72	0.029160	0.038978	0.009818	1.337
5	288	72	0.031298	0.036500	0.005202	1.166
**Overall Mean**			**0.029559**	**0.037677**	**0.008118**	**1.275**

Note: For each held-out fold, absolute SHAP values were averaged across patients and embedding dimensions separately for the 768-dimensional mean-pooled and 768-dimensional max-pooled representation halves. The max-pooled half showed a higher mean absolute contribution in all five folds. The overall mean was calculated as the arithmetic mean of the five fold-level summaries. For the overall row, the contribution ratio was calculated by dividing the overall mean absolute SHAP contribution of the max-pooled half by that of the mean-pooled half. The absolute-difference and contribution-ratio columns are descriptive quantities derived directly from the reported fold-level means. Because SHAP contribution magnitudes depend on feature scaling, model coefficients, and the distribution of the evaluated observations, these values should be interpreted as relative representation-level importance within the present model rather than as transferable biological effect sizes. SHAP, SHapley Additive exPlanations.

Supplementary Table S14. Fully nested candidate-pipeline selection sensitivity analysis.

The original five candidate pipelines were re-evaluated using inner patient-level cross-validation within each outer-training set. Candidate selection prioritized inner mean ROC-AUC and then inner mean PR-AUC; the selected pipeline was refitted on the complete outer-training set and evaluated once on the untouched outer-test fold.

Outer fold	Selected pipeline	Inner ROC-AUC	Inner PR-AUC	Outer ROC-AUC: selected	Outer PR-AUC: selected	Outer ROC-AUC: fixed LR	Outer PR-AUC: fixed LR
1	Logistic regression	0.904	0.782	0.930	0.815	0.930	0.815
2	Logistic regression	0.908	0.776	0.900	0.838	0.900	0.838
3	Logistic regression	0.921	0.803	0.863	0.808	0.863	0.808
4	Logistic regression	0.893	0.756	0.957	0.835	0.957	0.835
5	Logistic regression	0.908	0.800	0.926	0.751	0.926	0.751

Note: Logistic regression was selected in all five outer folds. Mean outer-fold ROC-AUC and PR-AUC were 0.915 ± 0.035 and 0.809 ± 0.035, respectively, for both the nested-selected pipeline and the fixed logistic-regression reference. Pooled out-of-sample ROC-AUC and PR-AUC were 0.915 and 0.802 for both approaches; paired bootstrap differences were exactly 0.000 for ROC-AUC and PR-AUC. This sensitivity analysis confirms reproducibility of the original pipeline selection within this cohort and does not establish intrinsic superiority of an algorithm family. LR, logistic regression; PR-AUC, area under the precision–recall curve; ROC-AUC, area under the receiver operating characteristic curve.

Supplementary Table S15. Logistic-regression regularization and class-weight sensitivity analysis.

Balanced and unweighted logistic-regression models were evaluated across C = 0.01, 0.1, 1, 10, and 100 using the same fixed five patient-level folds and within-fold preprocessing. This analysis was conducted as a sensitivity analysis; no configuration was selected retrospectively from these results.

Class weight	C	Mean ROC-AUC ± SD	Mean PR-AUC ± SD	Mean Brier ± SD	Pooled ROC-AUC	Pooled PR-AUC	Pooled Brier	Max iterations	Warnings
Balanced	0.01	0.923 ± 0.033	0.822 ± 0.059	0.077 ± 0.012	0.919	0.814	0.077	32	0
Balanced	0.1	0.917 ± 0.034	0.812 ± 0.044	0.073 ± 0.013	0.916	0.808	0.073	53	0
Balanced	1	0.915 ± 0.035	0.809 ± 0.035	0.076 ± 0.014	0.915	0.802	0.076	64	0
Balanced	10	0.903 ± 0.041	0.784 ± 0.042	0.084 ± 0.014	0.902	0.774	0.084	52	0
Balanced	100	0.892 ± 0.040	0.765 ± 0.053	0.114 ± 0.035	0.888	0.740	0.114	34	0
None	0.01	0.926 ± 0.033	0.832 ± 0.052	0.070 ± 0.012	0.921	0.823	0.070	38	0
None	0.1	0.918 ± 0.035	0.812 ± 0.045	0.071 ± 0.016	0.916	0.808	0.071	53	0
None	1	0.915 ± 0.036	0.805 ± 0.048	0.076 ± 0.017	0.914	0.800	0.076	61	0
None	10	0.909 ± 0.041	0.806 ± 0.054	0.080 ± 0.015	0.905	0.791	0.080	54	0
None	100	0.899 ± 0.033	0.783 ± 0.035	0.117 ± 0.031	0.897	0.761	0.117	29	0

Note: Stronger regularization (smaller C) produced similar or modestly higher internal discrimination than the prespecified C = 1 model, whereas weaker regularization reduced performance. At C = 1, removing class weighting yielded the same mean ROC-AUC (0.915) and a similar mean PR-AUC (0.805 versus 0.809). No convergence warnings occurred. Because this was not a hyperparameter-selection exercise, the prespecified balanced C = 1 logistic-regression model remained the primary model. PR-AUC, area under the precision–recall curve; ROC-AUC, area under the receiver operating characteristic curve; SD, standard deviation.

Supplementary Table S16. Clinicopathological and site-related associations with source-defined MSIMUT status.

Clinical and acquisition-site metadata linked from GDC were examined as internal sensitivity analyses to characterize potential outcome-associated covariates and site structure.

Variable	Statistical test	Available, n	Statistic	P value	Effect size	Effect-size measure
Age (years)	Mann-Whitney U	359	11869.500	0.0022	0.381	standardized mean difference (MSIMUT-MSS)
TCGA project	Chi-square with 5000 label permutations	360	16.375	0.0002	0.213	Cramer's V
Broad AJCC stage	Chi-square with 5000 label permutations	346	23.303	0.0002	0.260	Cramer's V
Tissue/organ of origin	Chi-square with 5000 label permutations	359	82.015	0.0008	0.478	Cramer's V
Primary diagnosis	Chi-square with 5000 label permutations	359	46.178	0.0006	0.359	Cramer's V
Tissue source site (TSS) code	Chi-square with 5000 label permutations	360	47.818	0.0540	0.364	Cramer's V

Note: All 360 patients were linked to GDC metadata. Age was available for 359 patients, broad pathologic stage for 346, tissue/organ of origin for 359, primary diagnosis for 359, and project/TSS for all 360. Sex was unavailable in the retrieved clinical metadata, and no clinical molecular-test MSI result was available. Categorical P values were obtained from chi-square statistics with 5,000 label permutations. The TSS association had a non-negligible effect size but did not reach the prespecified permutation-based significance threshold (P = 0.054). GDC, Genomic Data Commons; TSS, tissue source site.

Supplementary Table S17. Clinical-only, image-only, and image-plus-clinical model comparison.

Models were evaluated on the same patient-level folds to assess whether audited clinical variables explained or materially augmented the image-based signal.

Panel A. Cross-validated model performance

Model	Mean ROC-AUC ± SD	Mean PR-AUC ± SD	Mean Brier ± SD	Pooled ROC-AUC	Pooled PR-AUC	Pooled Brier
Clinical only	0.770 ± 0.086	0.433 ± 0.132	0.191 ± 0.013	0.761	0.374	0.191
Image only	0.915 ± 0.035	0.809 ± 0.035	0.076 ± 0.014	0.915	0.802	0.076
Image + clinical	0.916 ± 0.036	0.808 ± 0.047	0.076 ± 0.014	0.915	0.803	0.076

Panel B. Paired patient-level stratified bootstrap differences in pooled out-of-sample performance

Comparison	Δ ROC-AUC (95% CI)	Δ PR-AUC (95% CI)	Δ Brier (95% CI)
Image only − Clinical only	0.1533 (0.0818–0.2238)	0.4277 (0.2888–0.5248)	-0.1149 (-0.1444–-0.0850)
Image + clinical − Image only	0.0002 (-0.0025–0.0031)	0.0016 (-0.0059–0.0098)	-0.0007 (-0.0029–0.0013)
Image + clinical − Clinical only	0.1535 (0.0829–0.2230)	0.4293 (0.2923–0.5236)	-0.1156 (-0.1447–-0.0861)

Note: Clinical-only performance was lower than image-only performance. Adding the audited clinical variables to the image representation did not materially change pooled discrimination: image-plus-clinical minus image-only ΔROC-AUC = 0.0002 (95% CI, −0.0025 to 0.0031) and ΔPR-AUC = 0.0016 (95% CI, −0.0059 to 0.0098). Negative ΔBrier values favor the first-named model. CI, confidence interval; PR-AUC, area under the precision–recall curve; ROC-AUC, area under the receiver operating characteristic curve; SD, standard deviation.

Supplementary Table S18. Tissue-source-site-only and TSS-group-isolated sensitivity analyses.

Two complementary analyses were used to characterize the contribution of acquisition-site structure: prediction using TSS information alone and group-isolated validation in which TSS groups were separated across folds.

**Analysis**	**Mean ROC-AUC**	**SD ROC-AUC**	**Mean PR-AUC**	**SD PR-AUC**	**Mean Brier**	**Pooled ROC-AUC**	**Pooled PR-AUC**	**Pooled Brier**
TSS only	0.586	—	0.227	—	0.222	0.578	0.202	0.222
TSS-group-isolated image validation	0.867	0.047	0.714	0.101	0.110	0.849	0.645	0.117

Note: TSS-only performance was limited (mean ROC-AUC, 0.586; mean PR-AUC, 0.227). Image-model performance remained informative under TSS-group-isolated validation (mean ROC-AUC, 0.867 ± 0.047; mean PR-AUC, 0.714 ± 0.101), although it was lower than the standard patient-stratified internal validation. These analyses reduce concern that the reported image signal is explained solely by TSS structure but do not replace external multicenter validation. PR-AUC, area under the precision–recall curve; ROC-AUC, area under the receiver operating characteristic curve; TSS, tissue source site.

Supplementary Table S19. GDC-derived MSIsensor2 concordance and secondary-endpoint image-model performance.

GDC-derived MSIsensor2 metadata were used as a secondary audit of the source archive labels. This endpoint is bioinformatics-derived and was not treated as a uniformly clinically adjudicated MSI-H/dMMR reference standard.

Panel A. Concordance between source archive label and unambiguous GDC-derived MSIsensor2 status

**Measure**	**Value**
Total patients with MSIsensor2 metadata	360
Unambiguous MSI/MSS cases	351
Mixed/ambiguous cases	9
Agreement	98.9%
Cohen κ	0.959
Source-label sensitivity vs GDC status	0.983
Source-label specificity vs GDC status	0.990
Source-label PPV vs GDC status	0.950
Source-label NPV vs GDC status	0.997
TN / FP / FN / TP	290 / 3 / 1 / 57

Panel B. Existing image-model out-of-sample discrimination against the unambiguous GDC-derived MSIsensor2 endpoint

**Unambiguous, n**	**GDC MSI, n**	**GDC MSS, n**	**ROC-AUC**	**PR-AUC**	**Brier score**
351	58	293	0.917	0.798	0.071

Note: Agreement was calculated among the 351 patients with unambiguous GDC-derived MSI/MSS status; nine patients had mixed or ambiguous status across GDC files and were excluded from the concordance denominator. Four of the 351 unambiguous cases were discordant with the source archive label. The existing image-model out-of-sample probabilities retained strong discrimination against this secondary endpoint (ROC-AUC, 0.917; PR-AUC, 0.798). These results support label consistency but do not convert the source-defined endpoint into a clinically adjudicated assay-confirmed MSI-H/dMMR endpoint. GDC, Genomic Data Commons; MSI, microsatellite instability; MSS, microsatellite stable; NPV, negative predictive value; PPV, positive predictive value; PR-AUC, area under the precision–recall curve; ROC-AUC, area under the receiver operating characteristic curve.

Supplementary Table S20. Fixed-N and patch-count-adjusted sensitivity analyses addressing absolute patch-burden confounding.

A. Patch-count distribution and patch-count-only prediction

Analysis / Group	n	Mean patch count	SD	Median	IQR	Range	ROC-AUC	PR-AUC	Brier
MSS	295	397.5	530.2	243	97–458	6–3914	—	—	—
Source-defined MSIMUT	65	1154.4	989.7	854	452–1569	75–4305	—	—	—
Raw patch count, univariable	360	—	—	—	—	—	0.819	—	—
log1p patch count, univariable	360	—	—	—	—	—	0.819	—	—
Patch-count-only model, fixed five-fold CV	360	—	—	—	—	—	0.817	0.536	0.178

Note: Patch counts differed significantly between the source-defined outcome groups (Mann–Whitney U = 15,695.5, two-sided $p < 0.001$). The univariable ROC-AUC was identical for raw and log1p-transformed patch count because the logarithmic transformation is monotonic. The patch-count-only model was evaluated using the same fixed patient-level folds as the primary analysis.

B. Fixed-N sensitivity analysis compared with the same-cohort all-patch reference

Fixed N	Eligible n	Representation	Fixed-N ROC-AUC, mean ± SD	Fixed-N PR-AUC, mean ± SD	Fixed-N Brier, mean ± SD	Same-cohort all-patch ROC-AUC	Same-cohort all-patch PR-AUC	Same-cohort all-patch Brier
6	360	Mean	0.809 ± 0.024	0.590 ± 0.050	0.137 ± 0.014	0.820	0.592	0.141
6	360	Max	0.801 ± 0.029	0.579 ± 0.051	0.129 ± 0.012	0.912	0.767	0.085
6	360	Mean + Max	0.810 ± 0.025	0.596 ± 0.047	0.128 ± 0.013	0.915	0.809	0.076
25	345	Mean	0.824 ± 0.018	0.630 ± 0.029	0.137 ± 0.012	0.829	0.619	0.137
25	345	Max	0.808 ± 0.023	0.597 ± 0.038	0.129 ± 0.011	0.908	0.769	0.090
25	345	Mean + Max	0.831 ± 0.019	0.645 ± 0.037	0.123 ± 0.010	0.912	0.810	0.080
50	328	Mean	0.825 ± 0.009	0.635 ± 0.021	0.141 ± 0.005	0.825	0.635	0.140
50	328	Max	0.831 ± 0.023	0.647 ± 0.037	0.125 ± 0.012	0.903	0.766	0.094
50	328	Mean + Max	0.843 ± 0.016	0.680 ± 0.027	0.122 ± 0.007	0.908	0.810	0.084
100	280	Mean	0.815 ± 0.009	0.621 ± 0.014	0.157 ± 0.007	0.822	0.623	0.153
100	280	Max	0.830 ± 0.023	0.661 ± 0.048	0.140 ± 0.015	0.911	0.803	0.102
100	280	Mean + Max	0.838 ± 0.013	0.683 ± 0.029	0.137 ± 0.010	0.916	0.829	0.094
200	221	Mean	0.841 ± 0.004	0.738 ± 0.012	0.158 ± 0.004	0.841	0.735	0.158
200	221	Max	0.847 ± 0.010	0.752 ± 0.020	0.153 ± 0.010	0.900	0.823	0.120

Fixed N	Eligible n	Representation	Fixed-N ROC-AUC, mean ± SD	Fixed-N PR-AUC, mean ± SD	Fixed-N Brier, mean ± SD	Same-cohort all-patch ROC-AUC	Same-cohort all-patch PR-AUC	Same-cohort all-patch Brier
200	221	Mean + Max	0.854 ± 0.008	0.775 ± 0.011	0.146 ± 0.006	0.898	0.837	0.113

Note: Fixed-N patch sets were generated by random sampling without replacement and repeated 10 times for each N. Only patients with at least N available patches were eligible for the corresponding analysis. Because the eligible cohort changed as N increased, the all-patch comparator was recalculated separately for each N using exactly the same eligible patients and the same restricted original patient-level folds. Consequently, comparisons should be made within each N condition rather than directly across different N values.

C. Fold-contained patch-count residualization sensitivity analysis

Representation	Mean ROC-AUC	SD	Mean PR-AUC	SD	Mean Brier	Pooled ROC-AUC	Pooled PR-AUC
Mean only	0.820	0.046	0.592	0.076	0.141	0.820	0.566
Raw max only	0.912	0.036	0.767	0.062	0.085	0.909	0.761
Raw mean + max	**0.915**	0.035	**0.809**	0.035	**0.076**	**0.915**	**0.802**
Residualized max, linear	0.799	0.027	0.594	0.082	0.135	0.795	0.575
Mean + residualized max, linear	**0.898**	0.027	**0.745**	0.046	**0.093**	**0.899**	**0.727**
Residualized max, quadratic	0.767	0.042	0.559	0.125	0.145	0.765	0.549
Mean + residualized max, quadratic	**0.894**	0.031	**0.753**	0.050	**0.092**	**0.894**	**0.736**

Note: Patch-count residualization was fitted separately within each outer-training fold using log1p-transformed patient patch count and was then applied unchanged to the corresponding held-out patients. Linear and quadratic specifications were evaluated independently. No outer-test information was used to estimate the residualization relationship. These analyses were performed as sensitivity analyses and did not replace the prespecified primary model.

D. Patch-count dependence of max-pooled latent dimensions

Representation	Median absolute Spearman ϱ	Mean absolute Spearman ϱ	90th percentile	Maximum
Raw max-pooled dimensions	**0.411**	0.404	0.517	0.610
Linear residualized max	**0.023**	0.028	0.058	0.143
Quadratic residualized max	**0.014**	0.017	0.036	0.111

Note: Absolute Spearman correlations were calculated between patient patch count and each of the 768 max-pooled embedding dimensions. Values summarize the distribution of dimension-wise absolute correlations before and after fold-contained patch-count residualization.

Overall interpretation: Absolute patch burden was associated with the source-defined outcome and contributed materially to the predictive information carried by max-pooled representations. However, subsequent source-partition analyses showed that the apparent increment from max-containing representations was not stable under the reconstructed source holdout. These sensitivity analyses therefore indicate that patch burden contributes to the raw max-pooled signal and that residualized random-fold performance should not be interpreted as proving complete patch-count independence.

Supplementary Table S21. Source-partition holdout, explicit patch-count addition, and patch-count-decoding sensitivity analyses.

This table summarizes provenance-based internal sensitivity analyses based on reconstruction of the original source TRAIN/TEST partition. Source-TRAIN was used only for model fitting in the source-holdout analyses, and source-TEST was used once for evaluation. Bootstrap confidence intervals were calculated by class-stratified patient-level resampling of the source-TEST cohort unless otherwise specified. These analyses were internal source-structured sensitivity analyses and do not constitute independent external validation.

Panel A. Reconstructed source partition and patch-count distribution

Source partition	Source-defined class	Patients, n	Patches, n	Mean patches/patient	Median patches/patient	SD	Range
Source-TRAIN	MSIMUT	39	46,704	1,197.5	956.0	995.1	75–4,305
Source-TRAIN	MSS	221	46,704	211.3	165.0	171.4	6–862
Source-TEST	MSIMUT	26	28,335	1,089.8	700.5	997.7	80–4,064
Source-TEST	MSS	74	70,569	953.6	648.5	790.7	27–3,914

Panel B. Patch-count-only discrimination within reconstructed source partitions

Evaluation partition	Predictor	ROC-AUC	PR-AUC	Interpretation
Source-TRAIN	Raw patient patch count	0.918	0.815	Strong patch-count discrimination within source-TRAIN
Source-TEST	Raw patient patch count	0.517	0.330	Near-chance patch-count discrimination within source-TEST

Panel C. Source-TRAIN to source-TEST image-representation holdout performance

Representation	ROC-AUC	95% CI	PR-AUC	95% CI	Brier score	95% CI
Mean-only	0.7937	0.6705–0.8981	0.6676	0.5107–0.8434	0.1738	0.1112–0.2425
Max-only	0.7656	0.6554–0.8607	0.5480	0.4077–0.7158	0.2629	0.1946–0.3350
Mean-plus-max	0.7879	0.6793–0.8805	0.5933	0.4463–0.7671	0.2591	0.1886–0.3334

Panel D. Paired source-TEST bootstrap comparisons among representations

Comparison	ΔROC-AUC	95% CI	Interpretation
Mean-only minus mean-plus-max	0.0057	−0.0764 to 0.0852	No clear pairwise ROC-AUC difference
Mean-only minus max-only	0.0281	−0.0759 to 0.1289	No clear pairwise ROC-AUC difference
Mean-only minus patch-count-only	0.2768	0.0821 to 0.4626	Mean-only representation outperformed patch count in source-TEST

Panel E. Effect of explicitly adding patch count in the source-holdout setting

Model comparison	ROC-AUC without count	ROC-AUC with count	ΔROC-AUC	95% CI for ΔROC-AUC	PR-AUC without count	PR-AUC with count	Brier without count	Brier with count
Mean-only vs mean + patch count	0.7937	0.7957	0.0021	−0.0218 to 0.0244	0.6676	0.6472	0.1738	0.1710
Max-only vs max + patch count	0.7656	0.7640	−0.0016	−0.0078 to 0.0042	0.5480	0.5483	0.2629	0.2681
Mean-plus-max vs mean-plus-max + patch count	0.7879	0.7827	−0.0052	−0.0125 to 0.0000	0.5933	0.5877	0.2591	0.2645

Panel F. Count-only model in the source-holdout setting

Model	Source-TRAIN input	Source-TEST ROC-AUC	Source-TEST PR-AUC	Source-TEST Brier score
Count-only logistic regression	log1p-transformed patient patch count	0.5169	0.3301	0.4582

Panel G. Out-of-fold decoding of log1p-transformed patch count from raw and residualized max-pooled representations

Representation used for decoding	OOF R^2	Spearman ϱ	Spearman p-value	MAE for log1p patch count
Raw max-pooled representation	0.8742	0.9199	<0.0001	0.3467
Linear residualized max-pooled representation	−0.0123	−0.1314	0.0126	0.9880
Quadratic residualized max-pooled representation	−0.0123	−0.1314	0.0126	0.9880

Note: Source-TRAIN and source-TEST refer to the reconstructed original upstream partition, not to the random patient-level cross-validation folds used in the primary analysis. Patch-count-only discrimination was strong in source-TRAIN but near chance in source-TEST. Explicitly adding log1p-transformed patch count to the image-derived representations did not materially improve source-TEST discrimination. The patch-count-decoding audit showed that raw max-pooled embeddings strongly encoded patch count, whereas fold-contained residualization substantially attenuated useful multivariable patch-count decoding by R^2 and mean absolute error; however, a small inverse rank association remained after residualization. Linear and quadratic residualization produced numerically identical decoding summaries in the stored audit outputs. CI, confidence interval; MAE, mean absolute error; MSIMUT, source-defined microsatellite unstable or highly mutated category; MSS, microsatellite stable; OOF, out-of-fold; PR-AUC, area under the precision–recall curve; ROC-AUC, area under the receiver operating characteristic curve.

A hash-level audit of the retained array files confirmed that the linear and quadratic residualized max-pooled matrices were distinct. The linear and quadratic residualized max-pooled matrices were retained as separate arrays and were not numerically identical. In the retained decoding audit, however, their patch-count decoding summaries were identical to four decimal places for R^2, Spearman ϱ, p-value, and mean absolute error. Therefore, no independent interpretive distinction was made between the two residualization specifications in Panel G. These results were interpreted only as evidence that useful multivariable patch-count decoding was substantially attenuated by residualization, not as evidence that all patch-count-related information was completely removed.

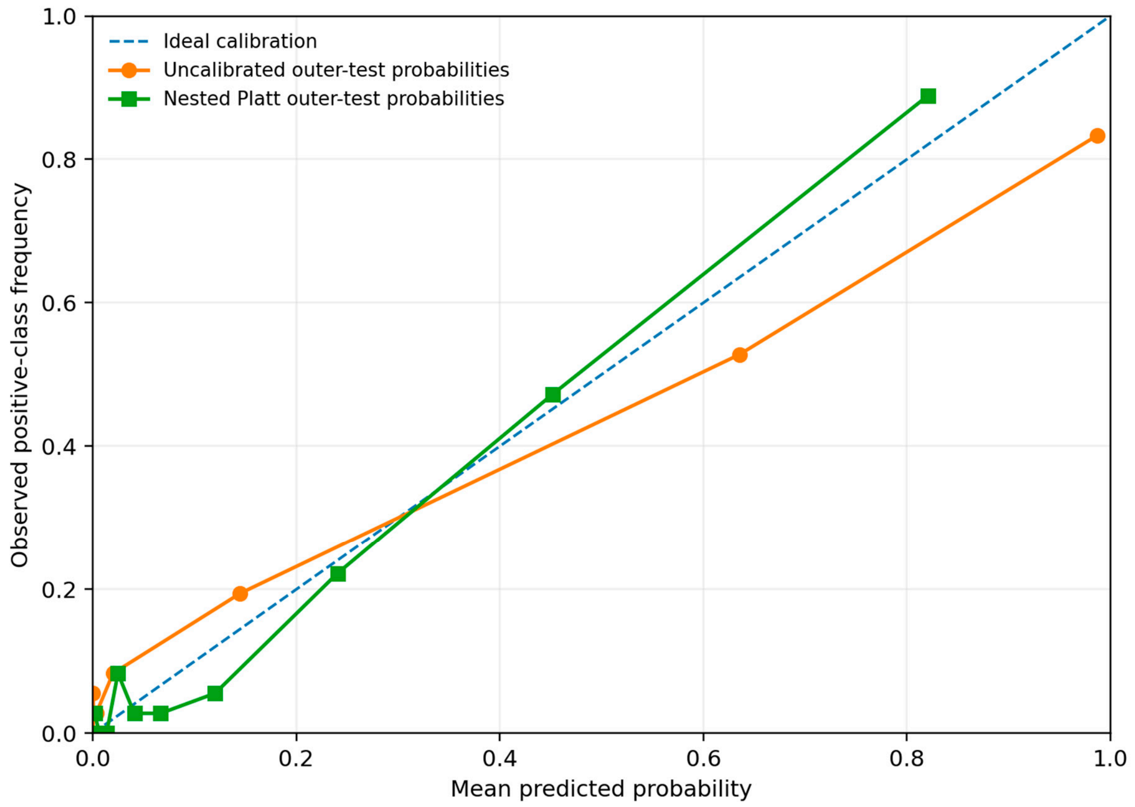

Figure S1. Reliability assessment of pooled outer-test probabilities before and after leakage-safe nested Platt calibration. For visualization, the mutually exclusive outer-test predictions were sorted separately for the uncalibrated and nested-Platt probability series and divided into 10 equal-sized patient groups (n = 36 per group). Each point shows the mean predicted probability and the observed source-defined positive-class frequency within that group. The diagonal dashed line represents perfect calibration. Calibration-method selection and fitting were performed within the nested procedure described in Supplementary Table S7; no outer-test outcome was used to select the calibration method.
